# A sequestered fusion peptide in the structure of an HIV-1 transmitted founder envelope trimer

**DOI:** 10.1038/s41467-019-08825-7

**Published:** 2019-02-20

**Authors:** Neeti Ananthaswamy, Qianglin Fang, Wadad AlSalmi, Swati Jain, Zhenguo Chen, Thomas Klose, Yingyuan Sun, Yue Liu, Marthandan Mahalingam, Subhash Chand, Sodsai Tovanabutra, Merlin L. Robb, Michael G. Rossmann, Venigalla B. Rao

**Affiliations:** 1 Department of Biology, The Catholic University of America, Washington, DC 20064 USA; 2 Department of Biological Sciences, Purdue University, West Lafayette, IN 47907 USA; 3 U.S. Military HIV Research Program, Henry M. Jackson Foundation for the Advancement of Military Medicine, Silver Spring, MD 20910 USA; 4 Present Address: The Fifth People’s Hospital of Shanghai & Institutes of Biomedical Sciences, Fudan University, Shanghai, 200032 People’s Republic of China

## Abstract

The envelope protein of human immunodeficiency virus-1 (HIV-1) and its fusion peptide are essential for cell entry and vaccine design. Here, we describe the 3.9-Å resolution structure of an envelope protein trimer from a very early transmitted founder virus (CRF01_AE T/F100) complexed with Fab from the broadly neutralizing antibody (bNAb) 8ANC195. The overall T/F100 trimer structure is similar to other reported “closed” state prefusion trimer structures. In contrast, the fusion peptide, which is exposed to solvent in reported closed structures, is sequestered (buried) in the hydrophobic core of the T/F100 trimer. A buried conformation has previously been observed in “open” state structures formed after CD4 receptor binding. The T/F100 trimer binds poorly to bNAbs including the fusion peptide-specific bNAbs PGT151 and VRC34.01. The T/F100 structure might represent a prefusion state, intermediate between the closed and open states. These observations are relevant to mechanisms of HIV-1 transmission and vaccine design.

## Abstract (web summary)

Here, the authors present the structure of the HIV envelope (Env) protein from a transmitted founder virus and show that, while the overall structure of the Env trimer is similar to other closed trimers, the fusion peptide is buried in the hydrophobic core of the trimer, which is similar to open state trimers.

## Introduction

Human immunodeficiency virus-1 (HIV-1) is the causative agent of acquired immunodeficiency syndrome or AIDS, a global pandemic affecting tens of millions of people worldwide. Despite more than 30 years of extensive research, an effective vaccine remains elusive. One of the principal targets for HIV-1 vaccine design is the trimeric envelope glycoprotein (Env) spike that is embedded in the viral envelope.

Env is synthesized as a heavily glycosylated gp160 protein precursor (gp refers to glycoprotein) and cleaved by the host furin protease to form a heterodimer (protomer) consisting of gp120 and gp41^1^ (Supplementary Fig. 1). Three such heterodimers form trimeric spikes on the viral membrane. The trimeric spikes facilitate entry of the virus into the host by a process that involves an intricate interplay between the spikes and the receptor molecules CD4 and CCR5 (or CXCR4) on the host cell, leading to viral and host cell membrane fusion and viral entry^2–4^.

The external part of the gp160 glycoprotein spike, gp140, has been successfully cloned, expressed, and purified^5,6^. Numerous structures of the gp140 trimer in the prefusion state have been determined by cryo-electron microscopy (cryo-EM)^7,8^ and X-ray crystallography^9^ at near-atomic resolution either when unliganded or when complexed with antibody fragments (Fabs), cellular receptors, and/or inhibitors^10–13^. All these structures, except four, correspond to the “closed” state in which the gp120 trimer wraps around the gp41 trimer, representing the virus before recognizing and fusing with a potential host.

Of the remaining four structures, the trimers in two are in an “open” state, whereas in the other two the trimer is in a “partially open” state^11,14^. In most of these structures, the open state is triggered by the binding of soluble CD4 (sCD4), the cell surface molecule that has been identified as a primary receptor for the HIV virus, and is stabilized by forming a complex with the Fab fragments of the CCR5 co-receptor binding antibodies 17b or 21c^11,14^. The open-state conformation thus represents the virus immediately prior to fusion with a host. Therefore, presumably, the open state must trigger membrane fusion and virus entry.

Here, we report the cryo-EM structure of an Env trimer isolated from a very early transmitted founder (T/F) virus and complexed with a Fab from the broadly neutralizing antibody (bNAb) 8ANC195. In this structure, the fusion peptide (FP) of the Env trimer is sequestered within the hydrophobic core of the trimer as was observed in the open state, while the rest of the trimer structure remains similar to the closed state. This Env trimer structure might represent a new prefusion state, in between the closed and open states that has not been previously described.

## Results

### Envelope trimers from an early transmitted founder virus

To produce trimers from a very early T/F virus, the Env genes were isolated by single genome amplification from the plasma of four high-risk volunteers (40007, 40061, 40094, and 40100) who participated in the RV217 Early Capture HIV Cohort Study conducted in Thailand^15^. The viruses belonged to the circulating recombinant form CRF01_AE. The participants were HIV-antibody and HIV-RNA negative, with a median of 4 days prior to becoming HIV-RNA positive, as determined by a sensitive nucleic acids test. Thus, the isolated Env sequences were from very recent infections, likely days before the samples were collected^15,16^ (Fiebig Stage 1). Alignment of multiple Env sequences, independently isolated from each individual, showed near 100% identity, even in the highly variable V1-V5 regions (Supplementary Fig. 1b, c), further confirming that a single T/F variant had established infection in each individual.

**Fig. 1:**
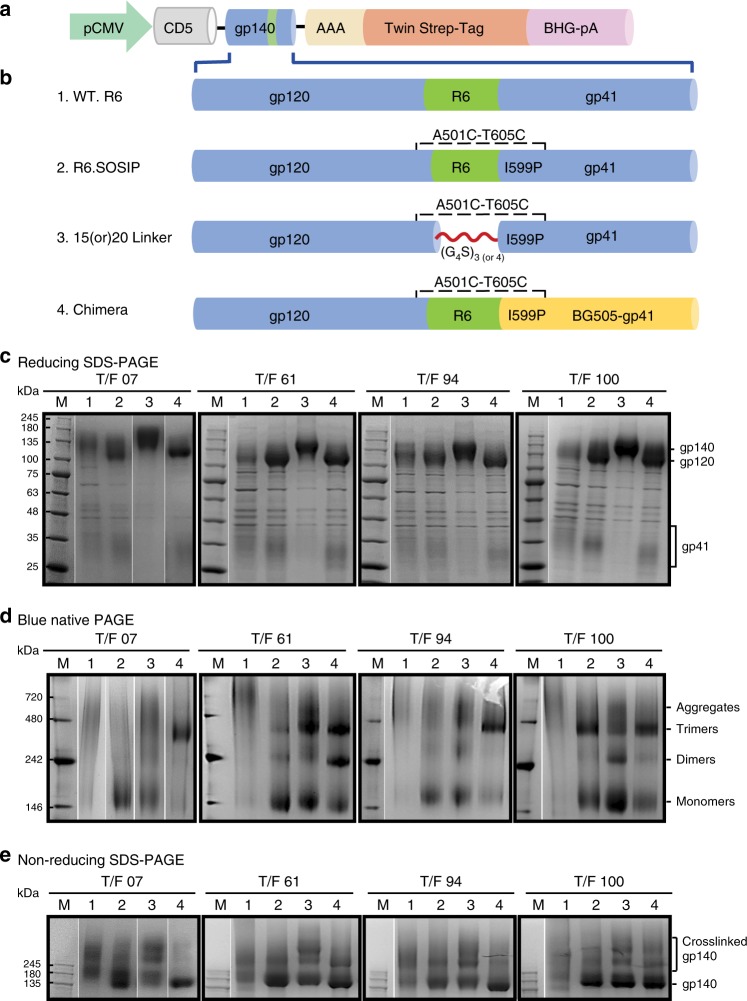
T/F gp140 recombinants predominantly produced gp140 monomers. **a** Schematic representation of gp140 Env expression vector. P_CMV,_ CMV promoter; CD5, human CD5 signal peptide; Twin Strep-Tag, the 31-amino-acid sequence attached to the C-terminus of gp140; BGH-_p_A, 3′ poly-A sequence of bovine growth hormone gene. **b** Schematic of gp140 recombinant constructs. (1) WT. R6, wild-type construct with a furin protease cleavage site RRRRRR (R6) between gp120 and gp41. (2) R6. SOSIP, the three SOSIP mutations A501C, T605C, and I599P introduced into the WT.R6 clone. (3) 15 (or) 20 Linker, 15 (G_4_S)_3_ or 20 (G_4_S)_4_ amino acid linker introduced between gp120 and gp41 replacing the furin cleavage sequence. This construct also includes the SOSIP mutations. (4) Chimera, BG505 gp41 sequence replaced the gp41 of CRF01_AE T/F gp140 sequence. This construct also includes the R6 cleavage site and the SOSIP mutations. **c**–**e** The gp140s secreted into the culture medium were purified by Strep-Tactin affinity chromatography and analyzed by SDS polyacrylamide gel electrophoresis (PAGE) under reducing (**c**) or non-reducing (**e**) conditions, and by blue native (BN) PAGE (**d**), and stained with Coomassie Blue. All the R6-gp140s were cleaved by furin as shown by the appearance of gp120 and gp41 bands (**c**). The WT gp140 aggregated, producing nonspecific oligomers and essentially no trimers (or protomers) (**d**, lanes 1). All the SOSIP gp140s predominantly produced monomers except the T/F100 gp140, which produced both monomers and trimers (**d**, lanes 2). Linker insertion prevented furin cleavage and increased trimer production except in the case of the T/F100 gp140 (**d**, lanes 3). A major shift to trimers was observed in all the three Chimera gp140s; T/F07, T/F61, and T/F94, but no significant difference was observed with the T/F100 (**d**, lanes 4). The Linker and Chimera trimers (**e**, lanes 3 and 4) showed greater aggregation and nonspecific crosslinking than the trimers lacking either the linker or the BG505 gp41 chimera (**e**, lanes 2), as evident by the presence of higher oligomer species under nonreducing conditions (**e**, compare lanes 2 to lanes 3 and 4). The molecular masses in kDa for lanes M are shown on the left

The four T/F gp140 Env sequences, hereafter referred to as T/F07, T/F61, T/F94, and T/F100, were cloned into a mammalian expression vector as described previously^17^ (Fig. 1). Among several features engineered into the recombinants (Fig. 1a, b), (i) the natural REKR furin cleavage site at the junction of gp120 and gp41 was replaced with an RRRRRR sequence to enhance cleavage, (ii) three “SOSIP” mutations; A501C, T605C, and I559P, were introduced to stabilize the cleaved trimers in a native-like conformation by disulfide crosslinking of gp120 and gp41 subunits^6,18,19^, and (iii) a twin Strep-tag with a 23-amino-acid flexible linker was fused to the C-terminus for affinity purification on a Strep-Tactin column^17^.

Notably, all the four T/F gp140 recombinants predominantly produced gp140 monomers (Fig. 1c–e; see lanes 2 in d). However, the T/F100 Env produced both monomers and trimers (Fig. 1d, lane 2). The T/F07, T/F61, and T/F94 gp140 trimers were then stabilized either by replacing the furin cleavage site with a 15- or 20-amino-acid linker (Linker trimers) (Fig. 1d, lanes 3) or by swapping the gp41 portion of the T/F gp140 Envs with the gp41 of BG505, which produces highly stable closed-state trimers (Chimera trimers) (Fig. 1d, lanes 4). Twelve different types of gp140 trimers, all having the same SOSIP mutations but differing in other features such as the presence of linker, length of linker, and presence of BG505 gp41, were then expressed and purified by Strep-Tactin affinity chromatography followed by size exclusion chromatography^17^ (Supplementary Fig. 2). A series of biochemical analyses (Supplementary Table 1, Supplementary Figs. 2 and 3) showed that the Linker and Chimeric trimers showed greater nonspecific crosslinking and were relatively heterogeneous when compared with the native-like trimers produced from the T/F100 virus. Furthermore, the T/F100 trimers produced in GNTI^−^ cells (high-mannose glycosylation) were more homogeneous than the trimers produced in 293 F cells (complex glycosylation) (Supplementary Fig. 3).

### Cryo-EM structure of the T/F100 Env trimer

**Fig. 2:**
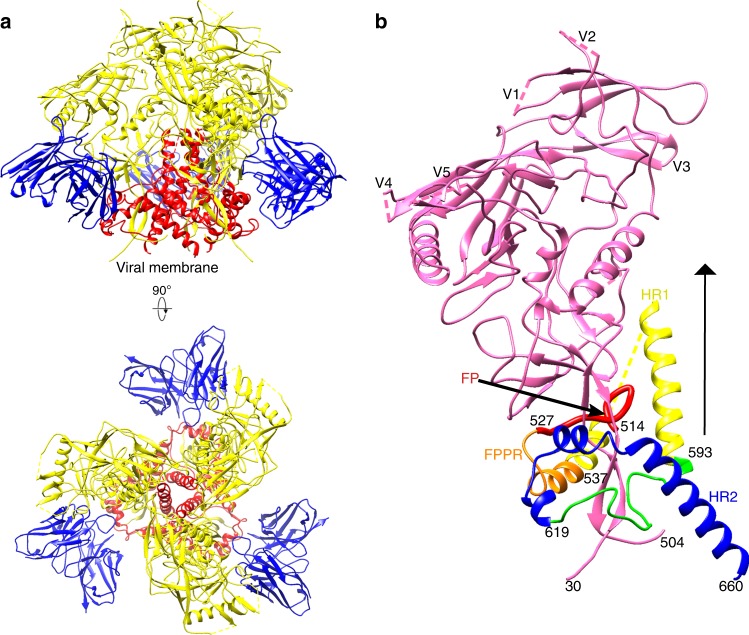
Structure of the T/F100 Env trimer in complex with Fab fragments of the bNAb 8ANC195**. a** Ribbon diagram of the atomic model of the T/F100 trimer in complex with the 8ANC195 Fab. Gp120, gp41, and the Fab are colored in yellow, red, and blue, respectively. Shown are the side view (top) and the view down the three-fold axis (bottom). **b** Ribbon diagram of a monomer of the T/F100 trimer. The three-fold axis is indicated by a black solid triangle. The fusion peptide (FP), the fusion peptide proximal region (FPPR), the heptad repeat 1 (HR1), and the heptad repeat 2 (HR2) regions of gp41 are colored red, orange, yellow, and blue, respectively. Gp120 is colored pink

Our initial attempts were to determine the cryo-EM structure of the unliganded T/F100 trimer. However, reference-free 2D classification showed similar three-bladed propeller shapes for most of the available particles, presumably representing the top view of the trimers, indicating severe orientation preference (Supplementary Fig. 4a). Therefore, the T/F100 trimers were investigated when incubated with different bNAbs, of which only the 8ANC195 antibody showed strong binding affinity (Supplementary Fig. 5). The T/F100 trimers complexed with 8ANC195 Fabs showed a more general distribution of orientation (Supplementary Fig. 4b). A de novo initial model was generated using the cryoSparc^20^ program assuming C3 symmetry, followed by using the program Relion^21^ to refine the structure to 3.9-Å resolution (Supplementary Figs. 4c–e and  6a, b). The final structure of the T/F100 trimer–8ANC195 Fab complex was similar to the closed structure of the previously determined BG505 trimer–8ANC195 complex^22,23^ (Fig. 2a). The T/F100 and BG505 Env molecules have an ~70% amino acid sequence identity. The root-mean-square deviation (rmsd) between equivalent Cα atoms of the superimposed structures was 1.8 Å. All the variable loops, except V3 on each gp120 monomer, were disordered in the earlier and in the present structure determinations (Fig. 2b).

The T/F100 trimer structure was also compared with 14 additional previously reported, closed-state structures, both unliganded trimers as well as trimers bound to various Fabs. The rmsd between equivalent Cα atoms of the superimposed structures ranged from 1.5 to 1.8 Å (Supplementary Table 2), indicating that the T/F100 trimer structure is in the closed conformation. Of the 30 potential N-linked glycosylation sites in the T/F100 gp140 sequence, there were 20 sites at which at least one sugar moiety was visible (Supplementary Fig. 7a, b). The remaining 10 sites were located mostly in the variable, disordered, loop regions, such as V1, V2, and V4. However, the T/F100 trimer structure has a glycan at N130 of gp120, unlike the other, previously determined, timer structures (Supplementary Fig. 7a–c). This glycan site is in the “A” β-strand of the V1/V2 region, which is a part of the epitope recognized by several bNAbs. Thus, this glycan might affect the binding of these bNAbs (see below).

### The T/F100 Env trimer has a sequestered fusion peptide

**Fig. 3:**
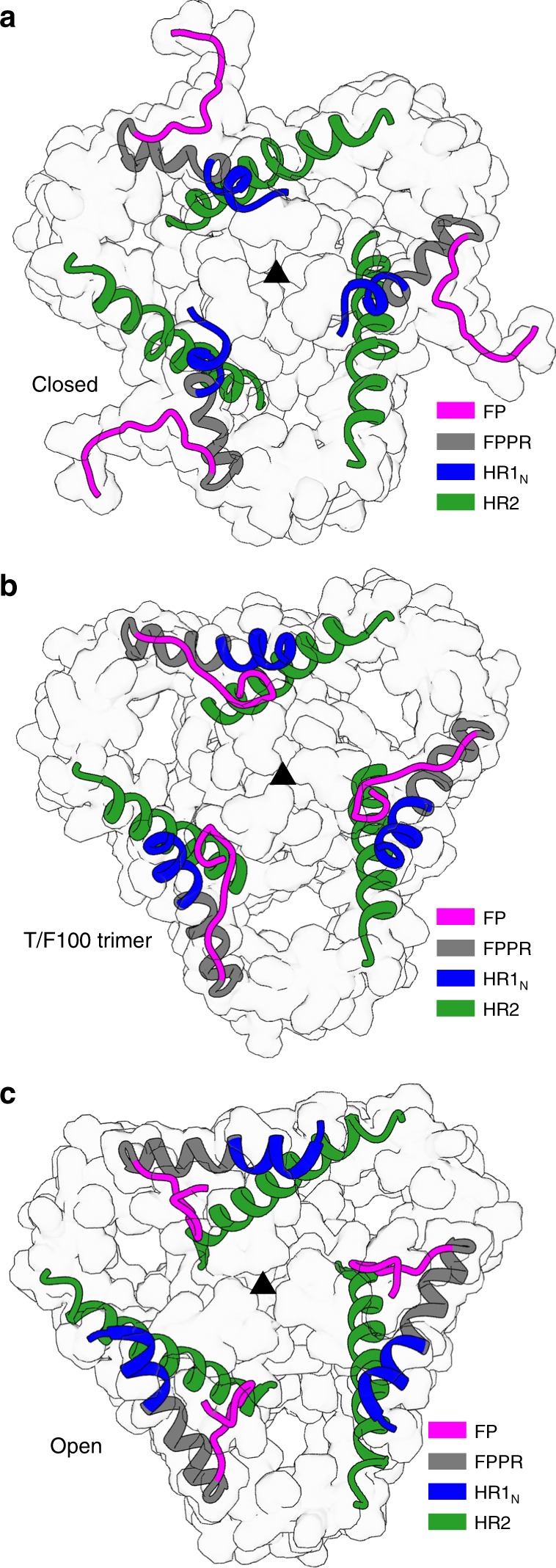
FP and HR1_N_ conformation of HIV-1 Env trimer structures. **a**–**c** FP and HR1_N_ conformation of **a** the closed trimer structure (PDB accession code 5I8H), **b** the T/F100 trimer structure and **c** the open trimer structure (PDB accession code 5VN3). Only gp41 of each trimer structure are shown for clarity. The FP, FPPR, HR1_N_, and the C-terminal part of HR2 (residues 637–664) regions of the trimer structures are shown as ribbon diagrams and semitransparent surfaces. Other parts of gp41 are shown only as surface representations. The three-fold axis of each structure is identified by a solid black triangle

Of particular significance were two structural differences between the T/F100 structure and the previously determined closed HIV-1 spike structures. The position of equivalent and ordered Cα atoms differed by more than 7 Å in the N-terminal part of the FP (residues 514–522) and in the N-terminal heptad repeat region 1 (HR1_N_) (residues 538–548) of gp41 (Fig. 3a, b). In the T/F100 trimer structure, almost all the residues of the FP were visible except for the first two residues (residues 512 and 513) (Supplementary Fig. 6c). Notably, the FP was deeply buried, with residues pointing toward the inner core of the trimer (Fig. 3b). Associated with this sequestered FP in the present structure was the repositioning of the HR1_N_ region to avoid steric clashes between the FP and the HR1_N_ region (Fig. 3a, b). Additionally, the HR1_N_ loop region forms an α-helix positioned along the fusion peptide proximal region (FPPR) (amino acids 530–547) packed against HR2 (Fig. 3b). Similar conformational rearrangements of the FP and HR1_N_ were observed in the open-state structures of HIV-1 Env trimer generated by binding the sCD4 receptor and the co-receptor binding antibody 17b^11^ (Fig. 3c). Similar buried conformation was also observed in the partially open-state trimer structures where the sCD4-opened BG505 trimers complexed with the Fabs of antibodies 17b or 21c were partially closed by the binding of the 8ANC195 Fabs^14^. In contrast to this buried FP conformation, the N-terminal nine residues of the FP in all the previously reported closed-state trimer structures are mostly disordered and exposed^24^ (Fig. 3a).

The HR1 loop of gp41 is an essential part of the proposed “spring loader” mechanism^25^, which, by changing to α-helix and lengthening the HR1 trimeric coiled coil, would propel the FP to the top of the gp41 structure and insert it into the host membrane. Considering that the densities of residues 514–520 of the FP are poor (Supplementary Fig. 6c), the FP of the T/F100 trimer might not have been well-stabilized and could move around the main chain. On the other hand, the FP of the open trimer structure was well-stabilized in a hydrophobic pocket formed by additional conformational rearrangements of the gp120 subunits as a result of binding to CD4 and the 17b antibody^11^. This is an important step in the membrane fusion reaction coordinate that eventually leads to irreversible transition into the fusogenic state^4,25^. The FP of the T/F100 trimer structure, unlike the other reported structures, is close to the residues that form this hydrophobic pocket in the open state. Further stabilization of the FP of the T/F100 trimer might require the formation of the hydrophobic pocket, presumably triggered by CD4 binding.

### T/F100 trimer bound poorly to Env and FP bNAbs

**Fig. 4:**
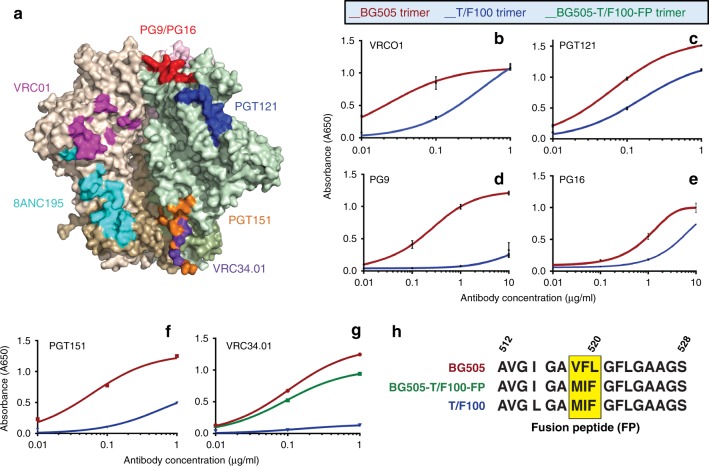
Antigenicity of the T/F100 trimer. **a** Epitope regions recognized by different bNAbs are shown on a surface-view structural model of BG505 gp140 trimer (PDB: 5I8H). Each protomer of the trimer are shown in different colors: brown, green, or pink. The gp120 and gp41 subunits of each protomer are shown in light and dark shades of the same color, respectively. **b**–**g** Binding curves of T/F100 (blue curve) and BG505 (red curve) trimers with bNAbs VRCO1 (**b**), PGT121 (**c**), PG9 (**d**), PG16 (**e**), PGT151 (**f**), and VRC34.01 (**g**), as determined by ELISA. Also shown in (**g**) is binding of BG505-T/F100-FP trimer to VRC34.01 (green curve). **h** Alignment of the fusion peptide sequences (amino acids 512–528) at the N-terminus of the gp41 subunit of BG505, BG505-T/F100FP, and T/F100 trimers. The three amino-acid residues that were swapped in BG505 trimer by the respective residues of T/F100 trimer to generate the BG505-T/F100FP mutant trimer are highlighted in yellow. ELISAs were performed using purified trimers coated on Strep-Tactin plates. Each binding curve was generated using data from three independent experiments. The trimer concentration was kept constant at 1 µg/ml and the bNAb concentration was varied as shown. Results shown are representative of at least three independent experiments performed in triplicates. The *P* value as determined by the unpaired two-tailed *t* test is 0.0006 for VRC01 at 0.1 μg/ml of antibody, < 0.0001 for PGT121, PG9 and PGT151, 0.0036 for PG16, and 0.0003 for VRC34.01 at 1 μg/ml of antibody. Error bars denote the standard deviation

The structural differences between the closed-state trimers and the T/F100 trimer as well as the presence of one additional glycan on the surface of the T/F100 trimer structure were further investigated using biochemical assays. The T/F100 trimer, unlike the spike structures of BG505 and other closed-state trimers, did not bind well to several potent bNAbs that recognize different epitopes on the trimer surface (Fig. 4a), including VRC01 (CD4 binding site; Fig. 4b), PGT121 (V3; Fig. 4c), PG9 (V1V2; Fig. 4d), and PG16 (V1V2; Fig. 4e).

A major difference between the T/F100 trimer and the BG505 trimer was the poor binding of T/F100 to the PGT151 bNAb (Fig. 4f). This is consistent with the buried conformation of the T/F100 FP because the PGT151 antibody binds across the boundary between gp120 and gp41 on the closed trimer surface^7,22^ and requires some of the N-terminal amino acids of the fusion peptide^7^, which in the case of the T/F100 trimer are not exposed.

To probe this point further, another FP-specific bNAb, VRC34.01, which has specificity for the first eight residues of the FP (amino acids 512–520), was examined when incubated with the T/F100 trimers. The residues recognized by VRC34.01 were found to be exposed in the closed-state trimer structures^26^, but not in the T/F100 trimer structure. The VRC34.01 antibody bound strongly to the closed trimer (BG505), whereas it showed negligible binding to the T/F100 trimer even at high antibody concentrations (10 µg/ml) (Fig. 4g).

Sequence alignment of the T/F100 and BG505 fusion peptides showed that 8 of the 12 N-terminal FP residues were identical and the remaining four residues had only conservative substitutions (Fig. 4h). To determine if these small sequence differences accounted for the lack of binding of the VRC34.01 antibody to T/F100 trimers, three of these amino acids that are in the FP binding site of the BG505 trimer were mutated to be the same as that of the T/F trimer (BG505-T/F100FP; Fig. 4h). The BG505-T/F100FP trimer showed only a small decrease in its binding ability to VRC34.01 (Fig. 4g), further confirming that the major difference in antibody binding was due to the antibody not being able to bind to the FP because it is buried in the T/F100 trimer.

## Discussion

Our studies identified a potentially new prefusion conformational state of the HIV-1 Env trimer that is in between the closed and open trimer states reported previously^8,9,11^. In this “intermediate” structure, the FP is buried and the proximal region of the FP and the contiguous N-terminal heptad 1 region form an α-helix. However, the rest of the Env trimer structure remains similar to the closed-state structures. We hypothesize that this conformation of the T/F100 virus Env trimer is at a step ahead of the closed state in the fusion pathway and might therefore provide an advantage over the viruses with closed-state trimers for membrane fusion and virus entry. Thus, this structure has implications for the mechanism of virus entry, nature of transmitted founder viruses, and vaccine design.

In the closed state of the HIV-1 trimer, the FP is exposed and disordered^24^. This has been observed in more than 15 prefusion closed-state trimer structures from different HIV-1 subtype viruses, both when unliganded and when complexed with various Fab molecules. This feature of the HIV FP is distinctly different from that found in the influenza virus, another type 1 fusion virus, and in all other well-characterized viral fusion proteins where the FP is sequestered in the envelope protein structure^25^. Therefore, the exposed HIV FP conformation could be considered an anomaly, and a consequence of SOSIP mutations and disulfide crosslinking of gp120 and gp41 subunits to stabilize the HIV trimer^6,18,19^. However, the same mutations and crosslinking resulted in a buried or sequestered conformation in the case of the T/F100 virus, which argues against this possibility. The buried conformation of T/F100 trimer was further confirmed by antibody binding. Antibodies such as PGT151 and VRC34.01 that recognize exposed FP showed dramatically decreased binding to the T/F100 trimer (Fig. 4f, g). Moreover, when the same SOSIP trimers with the exposed FP transition from the closed to open state^11^ or partially open state^14^ upon binding to a CD4 receptor, the conformation of the FP changes from an exposed conformation to a buried conformation. Alternatively, it could be argued that the buried conformation of T/F100 was due to a conformational change induced by binding of the 8ANC195 Fab. This is unlikely for the following reasons. (i) The 8ANC195 antibody binds equivalently to both the closed and open trimers, and its binding to closed trimer does not change the conformation significantly^22^. The structure of the closed-state BG505 trimer bound to the 8ANC195 Fab is similar to the other closed-state structures in which the trimers were bound to different Fabs^22,23,27–29^. (ii) With the exception of the FP region and the HR1N region, the overall structures of the 8ANC195-bound closed-state BG505 trimer and the 8ANC195-bound T/F100 trimer are similar to each other, which means no significant additional conformational change(s) occurred in the vicinity of the 8ANC195 binding site or elsewhere in the structure. This would have been expected if 8ANC195 were to induce a conformational change as was observed when 8ANC195 binding resulted in partial closing of the open trimers^14^. Thus, the evidence strongly suggests that the exposed FP conformation is indicative of the closed-state trimer and the buried FP conformation is indicative of the open-state trimer. The trimers from all the viruses reported thus far are in the closed state, whereas in the very early T/F100 founder virus studied here, they are in an intermediate state where the trimer has not opened but the FP conformational change has occurred.

The buried conformation of the T/F100 FP is accompanied by dynamic structural changes in the fusion machinery. The FPRR and the N-terminal sequence of heptad 1 form a longer α-helix and are repositioned. The FP moves closer to the residues in gp120 that eventually form a hydrophobic pocket in the open state. These are precisely the sort of changes that occur during the closed to open transition triggered by CD4 receptor binding^11,14^. Indeed, lengthening of the helix is an essential aspect of this conformational transition, when the relatively unstructured heptad 1 loop region in the prefusion state becomes a coiled coil in the fusogenic state^9^. Although the exact details vary, a similar transition also occurs in the Flu virus hemagglutinin (HA) trimer upon recognition of its host cell receptor^25^. These changes are part of the membrane fusion reaction coordinate that culminates in bringing the FP into close proximity to the host membrane facilitating FP insertion and membrane fusion^4,25^. Thus, the T/F100 fusion machinery might represent a new prefusion state, intermediate between the closed and open states. This might be a unique feature of the Env trimer from the CRF01_AE T/F100 HIV-1 strain used in this study and might represent a state in which the T/F100 trimer may have undergone some of the initial structural changes in and around the FP when the virus recognizes its host receptor and the membranes start to fuse.

An intermediate conformational state was inferred in previous single molecule FRET and mutational studies^30,31^. These studies showed that the HIV-1 trimer is an intrinsically dynamic molecule sampling three distinct conformations: closed, intermediate, and open. The closed state is the lowest energy state and the intermediate state is the highest energy state. Trimers transition from closed to open, or vice versa, through the intermediate state. The closed state is the most occupied state, however, trimers from different HIV-1 variants show different distributions of occupancy among the three states^31,32^. While the structures of closed and open states have been determined^9,11,28,32–35^, the intermediate state is controversial as there is no structural evidence. Although details vary, the Flu virus HA trimer also undergoes fluctuations between three analogous conformations^36^. Like in the case of HIV, the most open Flu trimer conformation is captured by interaction with its receptor, sialic acid, and the intermediate conformation lies in between similar closed and open conformations. The main difference between these states is the conformation of the FP. Whether the sequestered FP conformation captured in the T/F100 trimer is related to this FRET-based intermediate conformational state is unclear. The FRET intermediate trimers are reported to transition more efficiently into open state and require lower amounts of CD4 receptor than the trimers in the closed sate^30,31^. A leucine to alanine substitution at residue 193 of the V1V2 domain produced a virus with a higher occupancy of trimers in this intermediate state. The mutant virus was resistant to binding several broadly neutralizing antibodies including VRC01 and exhibited ~four-fold greater efficiency than the WT virus to infect human macrophages that contain very low amounts of the CD4 receptor^30,31,37^. These properties of the FRET intermediate conformational state appear to correlate with the poorly bNAb-reactive and predictably more fusion-prone intermediate structure described here. However, direct functional studies and additional trimer structures are needed to evaluate their significance.

During HIV-1 transmission, only one or a very few viral variants are successfully transmitted into the new host even though the host is likely exposed to billions of variants during sexual transmission^38^. Thus, a strong selection pressure exists against the passage of HIV through the mucosal barrier. While some of this selection might be stochastic, there is evidence that fitter variants get selected during this process. It is unknown as to what special signatures, if any, of the envelope protein of the transmitted founder viruses favor selection^38^. Persistence of an intermediate state envelope trimer in a very early transmitted founder virus described here might be significant. Particularly so, given that the T/F100 virus of participant 40100 was acquired by transmission through rectal mucosa that typically has very low abundance of the CD4 receptor^38^. Therefore, it is possible to speculate that the intermediate state trimers might impart a selective advantage for efficient transmission of the T/F100 virus through the mucosal barrier when compared with the vast majority of viruses in which the trimers are in the closed state. However, additional trimer structures from other T/F viruses and different HIV-1 subtypes are needed to determine if there is any functional linkage between the FP conformation and transmission efficiency.

Because the closed state is the most occupied state of trimers on the HIV-1 virions, they are considered to be the best immunogens for generating broadly neutralizing immunity^35^. The well-conserved FP that is exposed in the closed state is also considered to be a good target for stimulating bNAbs^26,39^. However, because only one or a few T/F viruses can successfully pass through the transmission bottleneck, the neutralizing immunity must be extremely strong to prevent entry of nearly all of the viruses. Moreover, a small minority of T/F viruses might still escape because, as our studies suggest, viruses with intermediate trimers would bind poorly to neutralizing antibodies that recognize the closed-state trimers. Therefore, it is essential to generate immunity against both the prefusion states, the closed state as well as the intermediate state, to block HIV infection. The intermediate Env trimer, thus, is a good candidate to include in HIV vaccine formulations, both for broadening the immune responses and perhaps for neutralizing the more transmission-competent T/F viruses.

## Methods

### Plasmids and clone constructions

Transmitted/founder (T/F) CRF01_AE HIV-1 gp160 Env DNA sequences (gp120 and gp41 ectodomain up to amino acid (aa) 664) from participants of the RV217-Early Capture Cohort Study [40007V02_01R, 40061V03_01 (GenBank ID: ASM58958.1), 40094V01_01R (GenBank ID: AML29865.1), 40100V01_01 (GenBank ID: ASM59319.1)]^16,40^ were codon optimized and the gene fragments were synthesized using GeneArt Strings technology (Life Technologies, Waltham, MA). The gp140 Env sequences of the codon optimized, cleavage-sensitive (R6) clones (40007V02_01R, 40061V03_01, 40094V01_01R, and 40100V01_01) are shown in Supplementary Table 3. Various mutations were introduced into the gp140 DNA by overlap extension PCR as described earlier^17,41^. The mutations include three SOSIP mutations A501C, T605C, and I599P^17,18,42^ and replacement of the natural furin cleavage sequence REKR with the enhanced cleavage sequence RRRRRR (R6)^43^, the 15- or 20-amino acid linker sequence (GGGGS)_3 or 4_^44,45^, or the cleavage resistant (CR) sequence SEKS^5,43,46^. Chimera clones have the SOSIP and R6 mutations plus the DNA sequence coding for BG505 gp41 instead of the T/F gp41^47^. Primers used to make these mutants are shown in Supplementary Table 4. The primers included tag sequences, which introduced *Nhe*I restriction site at the 5′ end and *Not*I restriction site at the 3′ end of the gp140 DNA sequence. This allowed directional cloning of the recombinant DNA into the plasmid vector. The mutant DNAs were inserted into the pcDNA3.1(−) vector such that the DNA is flanked by the CD5 signal peptide sequence corresponding to MPMGSLQPLATLYLLGMLVASV LA at the 5′ end (N-terminus) and the twin Strep-Tag II sequence corresponding to WSHPQFEK(GGGS)_2_GGSAWSHPQFEK at the 3′-end (C-terminus)^17^. Construction of the BG505 and JR-FL gp140 clones was described by AlSalmi et al.^17^. The furin-expressing plasmid, Furin:FLAG/pGEM7Zf( + ), was obtained from Gary Thomas (Vollum Institute, Portland, OR). The furin fragment from this plasmid was subcloned into pcDNA3.1(−) (Life Technologies, Inc.) using *Eco*RI and *Hind*III restriction sites. The accuracy of all the gp140 clones was confirmed by transformation into NEB 5-alpha Competent *E. coli* cells (New England Bio Labs, Inc., Ipswich, MA), purification of plasmid from positive clones by GeneJET plasmid miniprep kit (Life Technologies Carlsbad, CA), and DNA sequencing (Retrogen, Inc., San Diego, CA).

### Cells and media

HEK293F cells (Life Technologies, Catalog # R79007) were maintained in FreeStyle 293 expression media (Life Technologies). GnTI^−^ (HEK293S) cells (ATCC CRL-3022) were maintained in FreeStyle 293 expression media (Life Technologies) supplemented with 1% FBS (Quality Biologicals, Gaithersburg, MD). Cells were grown at 37 °C, 120 RPM, 80% humidity, and 8% CO_2_ in a Multitron Pro shaker (Infors HT, Bottmingen/Basel, Switzerland).

### Transfection

HEK293F cells were grown overnight in suspension to a density of 10^6^ cells/ml. Small-scale transfections were carried out in 100 ml cultures and large-scale transfections in 1 L cultures using the FreeStyle MAX reagent (Life Technologies). Briefly, the cells were transfected with 1 µg of gp140 plasmid DNA per 10^6^ cells. In the case of cleavage-sensitive Env clones (WT.R6, R6. SOSIP, Chimera), the cells were co-transfected with the furin plasmid at a gp140:furin plasmid DNA ratio of 3:1 to ensure near 100% cleavage. No furin plasmid DNA was co-transfected in the case of Linker or CR clones. The plasmid DNAs were mixed with the MAX reagent diluted in OptiPRO SFM medium and incubated for 10 min at room temperature. The mixture was then added to the HEK293F suspension cells. After 12 h, 1 mM sodium butyrate (Sigma-Aldrich, St. Louis, MO) and 10% HyClone SFM4HEK293 media (GE Healthcare, Logan, UT) were added to the cells. The transfected cultures were incubated at 37 °C, 80% humidity, 8% CO_2_, and 120 RPM for 5 days.

Transfection of the GnTI^−^ (HEK293S) cells was performed using the polyethylenimine reagent (PEI 25k; Polysciences, Inc., Warrington, PA)^17^. Briefly, cultures were centrifuged at 100 *g* for 5 min at 37 °C and resuspended in fresh FreeStyle 293 expression media to a density of 2 × 10^6^ cells/ml. Various gp140 plasmid DNAs were co-transfected with the furin plasmid at a gp140:furin plasmid DNA ratio of 3:1. No furin plasmid DNA was co-transfected in the case of the Linker or CR gp140 plasmid DNAs. About 1 µg plasmid DNA per 10^6^ cells was added to the culture followed by the addition of PEI at a PEI:DNA (w/w) ratio of 3:1. After 4 h of incubation, 2 mM sodium butyrate (Sigma-Aldrich, St. Louis, MO) and 50% HyClone SFM4HEK293 (GE Healthcare) were added. The transfected cultures were incubated at 37 °C, 80% humidity, 8% CO_2_, and 120 RPM for 5 days.

### Purification of gp140

Culture supernatants containing the secreted gp140 proteins were harvested at day 5 by centrifugation at 3,000 × *g* for 10 min at 4 °C. Supernatants were then vacuum filtered using 0.2 µm filters (Corning, Inc.). Protease inhibitor cocktail tablets (Roche Diagnostics) and BioLock biotin blocking solution (iba Life Sciences) were added to the cultures and incubated at 4 °C for 30 min. The sample was loaded on a Strep-Tactin column (Qiagen) using the ÄKTAprime plus liquid chromatography system (GE Healthcare). Wash buffer (50 mM Tris-HCl, pH 8.0, 300 mM NaCl) was then passed through the column to remove the unbound contaminants. The twin Strep-Tag II^−^gp140 Env bound to the Strep-Tactin column was eluted with a buffer containing 50 mM Tris-HCl, pH 8.0, 300 mM NaCl, and 2.5 mM d-Desthiobiotin. The purified gp140 protomers containing monomers, dimers, and trimers were then separated by size exclusion chromatography using the Hi-Load 16/600 Superdex-200 (prep-grade) gel filtration column (AKTA FPLC, GE Healthcare) equilibrated with a buffer containing 25 mM Tris-HCl, pH 8 and 300 mM NaCl. Selected fractions were concentrated by Amicon centrifugal filtration (100 kDa cut-off) and stored at −80 °C in the presence of 10% glycerol. Additional details of the purification protocol were described in AlSalmi et al.^17^. See Fig. 1 and Supplementary Figs. 2 and 3 for details on the composition of various purified trimer preparations. All uncropped protein gels are shown in a supplementary figure in the Supplementary Information (Supplementary Fig. 8).

### Antigenicity studies using Strep-Tactin ELISA

ELISAs were performed on Strep-Tactin-coated 96-well microtiter plates (iba Life Sciences) according to the protocol described by AlSalmi et al.^17^. Strep-Tactin-coated microplates (IBA Life Sciences) were coated with 1 µg/ml SEC-purified gp140 trimers for 2 h at RT, and washed thrice with PBS. This procedure allowed proper orientation of the trimer, base attached to the plate and apex and rest of the trimer exposed to the antibody. Serially diluted antibodies (0.001, 0.1, 1.0, and 10 µg/ml) were added to the wells, and incubated for 1 h at 37 °C. After three washes with PBST (0.05% Tween 20 in PBS), the plates were incubated with rabbit antihuman antibody HRP conjugate (Santa Cruz Biotechnology, Catalog No. SC2769) diluted 1:3,000 in PBS for 30 min at 37 °C. The plates were washed three times with PBST, and the peroxidase substrate was added to develop the color reaction (TMB microwell peroxidase substrate system, KPL). The reaction was terminated by adding BlueSTOP solution (KPL), and OD650 was recorded using the VersaMax ELISA plate reader (Molecular Devices, Sunnyvale, CA). The data were plotted using the Graphpad Prism software (La Jolla, CA). The *P* value as determined by the unpaired two-tailed *t* test is 0.0006 for VRC01 at 0.1 μg/ml of antibody, <0.0001 for PGT121, PG9 and PGT151, 0.0036 for PG16, and 0.0003 for VRC34.01 at 1 μg/ml of antibody. Error bars denote standard deviation. Immobilization of the trimer through the Strep-tag linker on the Strep-Tactin plate in PBS buffer allowed trimers to be displayed in a proper orientation with minimal effect on structural integrity. Also, the flexible linker provided enhanced access for binding of the antibody to the trimer. The bNabs PG9^48^ and PG16^48^ that recognizes epitopes on V1V2 and the interface antibody PGT151^49^ were obtained from Scripps Research Institute and International AIDS Vaccine Initiative Neutralizing Antibody Center (IAVI NAC). The bNAbs VRCO1^50^ (Catalog No. 12033), a CD4 binding site-specific antibody and PGT121^51^ (Catalog No. 12343), a V3 site-specific antibody were obtained from the NIH AIDS Reagent Program, Division of AIDS, NIAID, NIH. The gp120- gp41 interface antibody 8ANC195 Fab was kindly provided by Pamela Bjorkman, (California Institute of Technology, CA). FP-specific bNAb, VRC34.01^26^, was kindly provided by Peter Kwong (Vaccine Research Center, NIH).

### Assembly of T/F100 Env trimer–8ANC195 Fab complex

To assemble the T/F100 Env trimer–Fab complex for cryo-EM, three-fold molar excess of 8ANC195 G52K5 Fab (kindly provided by Pamela Bjorkman, California Institute of Technology, CA) was mixed with T/F100 Env trimer produced in GNTI^−^ cells and incubated at room temperature for 12 h. The complex was then purified on a size exclusion column (Superdex-200, GE Healthcare) equilibrated in the gel filtration buffer (20 mM Tris-HCl, pH 8, 100 mM NaCl) using the NGC chromatography system (Bio-Rad, Hercules, CA). Protein peak corresponding to the T/F100 Env trimer–8ANC195 Fab complex, as evident from SDS-PAGE analysis, was used for cryo-EM (Supplementary Fig. 5). The uncropped SDS-PAGE gel of T/F100 trimer–8ANC195 Fab complex is provided as a supplementary figure in the Supplementary Information (Supplementary Fig. 8).

### Cryo-electron microscopy

Samples of the T/F100 Env trimer–8ANC195 Fab complex were prepared for cryo-EM by mixing dodecyl maltoside (DDM) with the complex to give a final DDM concentration of 0.085 mM^8^. Aliquots of 2.7 µl of either the unliganded T/F100 trimer or the T/F100 trimer–8ANC195 Fab complex were applied to Lacey carbon EM grids. The grids were blotted for 6 s and flash frozen in liquid ethane using a Gatan CP3 freezer. The frozen grids were loaded into an FEI Titan Krios EM, operated at 300 kV. The microscope was equipped with a Gatan K2 Summit detector. The data were collected with Leginon^52^ using a nominal magnification of ×29,000 with a dose rate of ~8 e^−^/(pixel·s) in the “super-resolution” mode, resulting in a pixel size of 0.5 Å. For the data set of the T/F100 trimer–8ANC195 Fab complex, each micrograph was recorded as a movie composed of 40 frames with an exposure time of 200 ms per frame and a total dose of ~64.0 e^−^/Å^2^. A total of 844 such movies were collected. For the data set of the unliganded T/F100 trimer, each micrograph was recorded as a movie composed of 25 frames with an exposure time of 200 ms per frame and a total dose of ~40.0 e^−^/Å^2^. A total of 2356 such movies were collected.

### Image processing of the T/F100 Env trimer–8ANC195 Fab complex

The 40 frames of each micrograph of the T/F100-8ANC195 Fab complex data set were aligned using the MotionCorr program^53^. For each aligned movie, all the 40 frames except for the first frame were used to produce an averaged micrograph. The CTFFIND3 program^54^ was used to estimate the contrast transfer function parameters for the averaged micrographs. To accelerate the computation process, the averaged micrographs were binned by a factor of 2, resulting in a pixel size of 1.0 Å. Particle picking, 2D classification, 3D refinement, and 3D classification were performed with the RELION-1.4 software^21^. Around 1000 manually picked particles were used to calculate templates for reference-based particle picking. Around 353,000 particles were picked automatically using templates, which had been low-pass filtered to 20 Å to limit reference bias. After two rounds of 2D classification, 170,716 particles were selected for further data processing.

To compensate for the radiation damage, the particles used in 2D classification were re-boxed from the aligned movies using only frames 2–11. The anisotropic magnification of the particles was corrected with the JSPR program^55,56^. The 3D refinement was initiated with a 60 Å low-pass filtered model generated from cryoSPARC (ab-initio reconstruction)^20^ using all the particles selected by 2D classification. C3 symmetry was imposed during the process of 3D refinement. This gave a 3.9 Å resolution reconstruction. The reported resolution was estimated in RELION using the gold-standard Fourier shell correlation (FSC = 0.143) criterion^57^ by applying a soft mask to both the two half maps. An attempt to perform 3D classification with fixed alignment parameters obtained from the 3D refinement did not improve the cryo-EM map. In addition, the 3D classification did not result in any reconstruction that showed different conformations when compared with the reconstruction obtained in the 3D refinement.

### Image processing of unliganded T/F100 Env trimer data set

The 25 frames of each micrograph were aligned using the MotionCorr program^53^. For each aligned movie, all the 25 frames except for the first frame were used to produce an averaged micrograph. The CTFFIND3 program^54^ was used to estimate the contrast transfer function parameters for the averaged micrographs. To accelerate the computation Process, the averaged micrographs were binned by a factor of 2, resulting in a pixel size of 1.0 Å. Particle picking and 2D classification were performed with the RELION-1.4 software^21^. Around 180,000 particles were picked following the same procedure used to pick the particles of the T/F100 Env trimer–8ANC195 Fab complex as described above.

### Model building and refinement

Initial models of T/F100 gp120 and gp41 were generated with Swiss-modeling^58–60^, using the structure of the JR-FL SOSIP.664 trimer in complex with the broadly neutralizing antibodies PGT122, 35O22, and VRC01 (PDB: 5FYK) as a template. The X-ray crystal structure of the variable domains (PDB: 5CJX) of 8ANC195 Fab and the initial models of T/F100 gp120 and gp41 were manually fitted into the cryo-EM map with the Chimera program^61^ and rebuilt in Coot^62^. Because the T/F100 Env trimer was expressed in GNTI^−^ cells, high mannose sugars were built into the electron densities of the N-linked glycans^63^. Because the local resolution of the region representing the constant domains of the 8ANC195 Fab was poor, no attempt was made to build structure into this density. The model was subsequently subjected to real-space refinement in Phenix^64^ (Supplementary Table 5). The programs MolProbity^65^, pdb-care^66^, and CARP^67^ were used to analyze the quality of the final model.

### Reporting summary

Further information on experimental design is available in the Nature Research Reporting Summary linked to this article.

## Supplementary information

Reporting Summary

Supplementary Information

## Data Availability

The cryo-EM reconstruction of the T/F100 trimer in complex with Fab fragments of the bNAb 8ANC195 has been deposited in the Electron Microscopy Data Bank under the accession number EMD-0485. The atomic model of the T/F100 trimer in complex with Fab fragments of the bNAb 8ANC195 has been deposited in the Protein Data Bank under accession number 6NQD.
