# Supplementary material for: A sequestered fusion peptide in the structure of an HIV-1 transmitted founder envelope trimer
Source: Nat Commun. 2019 Feb 20;10:873. doi: 10.1038/s41467-019-08825-7 (PMC6382815; doi:10.1038/s41467-019-08825-7)
Supplement: Supplementary file 1 — Reporting Summary [file 41467_2019_8825_MOESM1_ESM.pdf]

nature research

Corresponding author(s): Michael G. Rossmann
Venigalla B. Rao

Reporting Summary

Nature Research wishes to improve the reproducibility of the work that we publish. This form provides structure for consistency and transparency in reporting. For further information on Nature Research policies, see Authors & Referees and the Editorial Policy Checklist.

Statistical parameters

When statistical analyses are reported, confirm that the following items are present in the relevant location (e.g. figure legend, table legend, main text, or Methods section).

n/a	Confirmed	
☐	☒	The exact sample size (n) for each experimental group/condition, given as a discrete number and unit of measurement
☐	☒	An indication of whether measurements were taken from distinct samples or whether the same sample was measured repeatedly
☐	☒	The statistical test(s) used AND whether they are one- or two-sided<br>*Only common tests should be described solely by name; describe more complex techniques in the Methods section.*
☒	☐	A description of all covariates tested
☒	☐	A description of any assumptions or corrections, such as tests of normality and adjustment for multiple comparisons
☒	☐	A full description of the statistics including central tendency (e.g. means) or other basic estimates (e.g. regression coefficient) AND variation (e.g. standard deviation) or associated estimates of uncertainty (e.g. confidence intervals)
☒	☐	For null hypothesis testing, the test statistic (e.g. F, t, r) with confidence intervals, effect sizes, degrees of freedom and P value noted<br>*Give P values as exact values whenever suitable.*
☒	☐	For Bayesian analysis, information on the choice of priors and Markov chain Monte Carlo settings
☒	☐	For hierarchical and complex designs, identification of the appropriate level for tests and full reporting of outcomes
☒	☐	Estimates of effect sizes (e.g. Cohen's d, Pearson's r), indicating how they were calculated
☐	☒	Clearly defined error bars<br>*State explicitly what error bars represent (e.g. SD, SE, CI)*

Our web collection on statistics for biologists may be useful.

Software and code

Policy information about availability of computer code

Data collection	No new softwares. Published softwares are listed with references in Methods.
Data analysis	leginon, jspr, phenix, chimera, MotionCorr 2.0, CTFFIND3, Relion 1.4, Coot

For manuscripts utilizing custom algorithms or software that are central to the research but not yet described in published literature, software must be made available to editors/reviewers upon request. We strongly encourage code deposition in a community repository (e.g. GitHub). See the Nature Research guidelines for submitting code & software for further information.

Data

Policy information about availability of data

All manuscripts must include a data availability statement. This statement should provide the following information, where applicable:
- Accession codes, unique identifiers, or web links for publicly available datasets
- A list of figures that have associated raw data
- A description of any restrictions on data availability

Atomic coordinates and the electron density map have been deposited to PDB and EMDB, respectively. They will be made available upon publication.

April 2018

Field-specific reporting

Please select the best fit for your research. If you are not sure, read the appropriate sections before making your selection.

☒ Life sciences ☐ Behavioural & social sciences ☐ Ecological, evolutionary & environmental sciences

For a reference copy of the document with all sections, see nature.com/authors/policies/ReportingSummary-flat.pdf

Life sciences study design

All studies must disclose on these points even when the disclosure is negative.

Sample size	N/A
Data exclusions	No data were excluded from analyses.
Replication	As stated in the manuscript, independent experiments were performed to reproduce the results and the results were successfully reproduced.
Randomization	N/A
Blinding	N/A

Reporting for specific materials, systems and methods

Materials & experimental systems

n/a	Involved in the study
☐	☒ Unique biological materials
☐	☒ Antibodies
☐	☒ Eukaryotic cell lines
☒	☐ Palaeontology
☒	☐ Animals and other organisms
☒	☐ Human research participants

Methods

n/a	Involved in the study
☒	☐ ChIP-seq
☒	☐ Flow cytometry
☒	☐ MRI-based neuroimaging

Unique biological materials

Policy information about availability of materials

Obtaining unique materials	All unique materials are readily available from the authors or from standard commercial sources as specified in the manuscript.

Antibodies

Antibodies used	The sources of all the antibodies used in the study are specified in Methods.
Validation	All the antibodies used in the study are highly purified reagents. The functionality of the antibodies was validated by several assays such as ELISA and virus neutralization, as well as structure determination. The relevant information and references are included in the manuscript.

Eukaryotic cell lines

Policy information about cell lines

Cell line source(s)	Standard commercial sources for the cell lines used are specified in Methods.
Authentication	N/A
Mycoplasma contamination	The cell lines were not tested for mycoplasma contamination after receiving them from commercial sources as specified in Methods.

Commonly misidentified lines<br>(See ICLAC register)	N/A
