# Supplementary material for: A sequestered fusion peptide in the structure of an HIV-1 transmitted founder envelope trimer
Source: Nat Commun. 2019 Feb 20;10:873. doi: 10.1038/s41467-019-08825-7 (PMC6382815; doi:10.1038/s41467-019-08825-7)
Supplement: Supplementary file 2 — Supplementary Information [file 41467_2019_8825_MOESM2_ESM.pdf]

A sequestered fusion peptide in the structure of an HIV-1 transmitted founder envelope trimer

Supplementary Information

Neeti Ananthaswamy, Qianglin Fang, Wadad AlSalmi, Swati Jain et al.

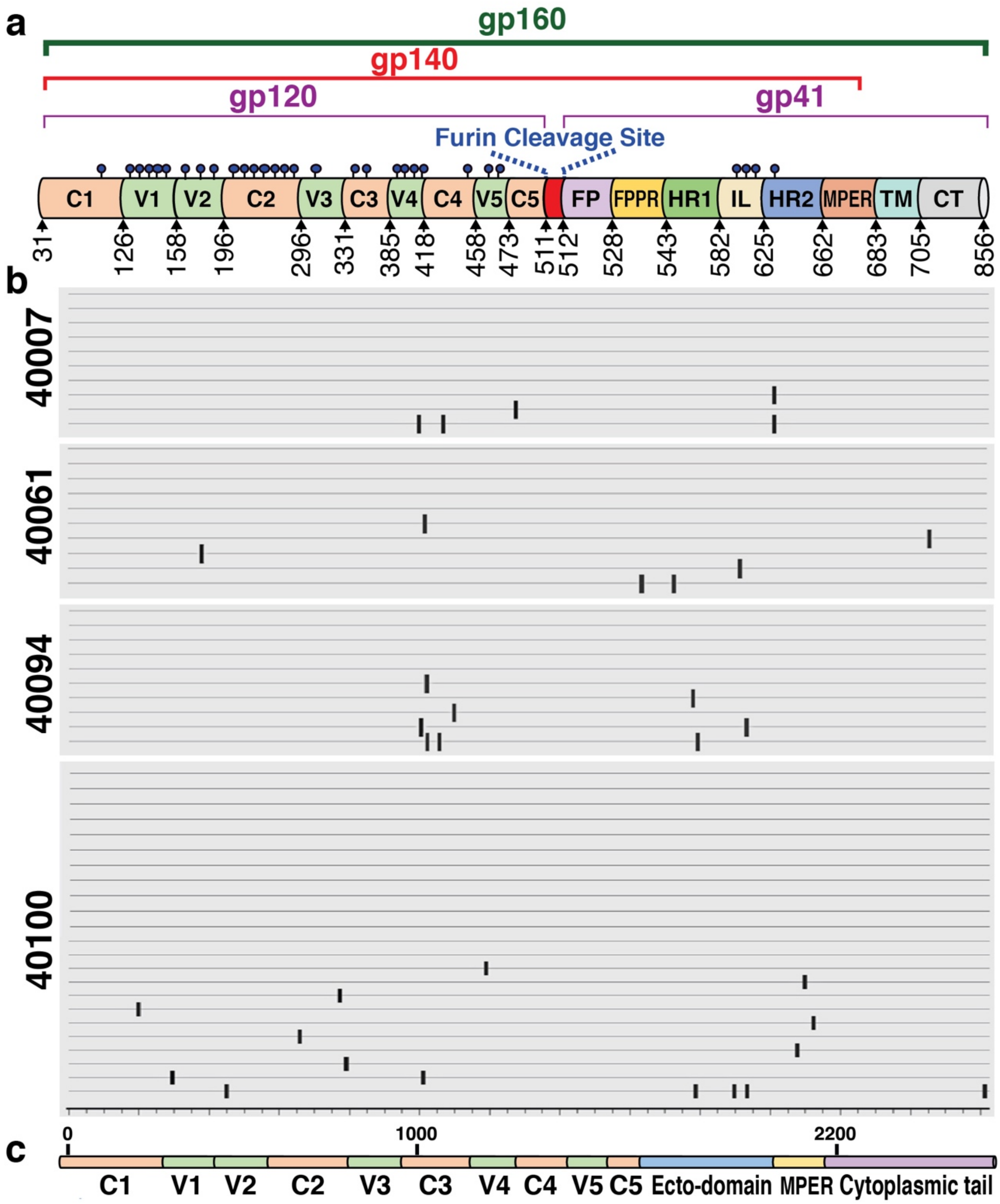

Supplementary Figure 1. Schematic representation of HIV-1 envelope proteins. **a** The HIV-1 envelope glycoprotein (Env) is synthesized as a 160 kDa precursor and cleaved by furin protease to produce the heterodimer (protomer) consisting of gp120 and gp41. gp140 represents the external section of the envelope proteins that includes gp120 and gp41 up to the transmembrane domain (TM). gp120 contains five conserved regions (C1–C5) and five variable regions (V1–V5). gp41 contains the fusion peptide (FP) at the N-terminus, the fusion

peptide proximal region (FPPR), the heptad repeat-1(HR-1), the immune dominant loop (IL), the heptad repeat-2 (HR-2), MPER (membrane proximal external region) and CT (cytoplasmic tail). The potential N-linked glycosylation sites are shown as blue tree symbols. The numbers correspond to the number of the amino acid at that position of the gp160 coding sequence. The N-terminal 30 amino acids that correspond to the signal peptide are not shown because these are replaced with the human CD5 signal peptide in all the gp140 clones. **b** Alignment of Env nucleotide sequences from participants of the RV217 study. Each horizontal line represents one gp160 sequence amplified from the plasma of participants from the RV217 Early Capture HIV Cohort Study by single genome amplification and sequenced. The Highlighter software is used to align the sequences. Each short vertical line represents a single mutation at that position of the aligned sequence relative to the most common sequence from the respective participant. **c** Schematic representation of the gp160 Env polypeptide aligned with the corresponding nucleotide sequences shown above.

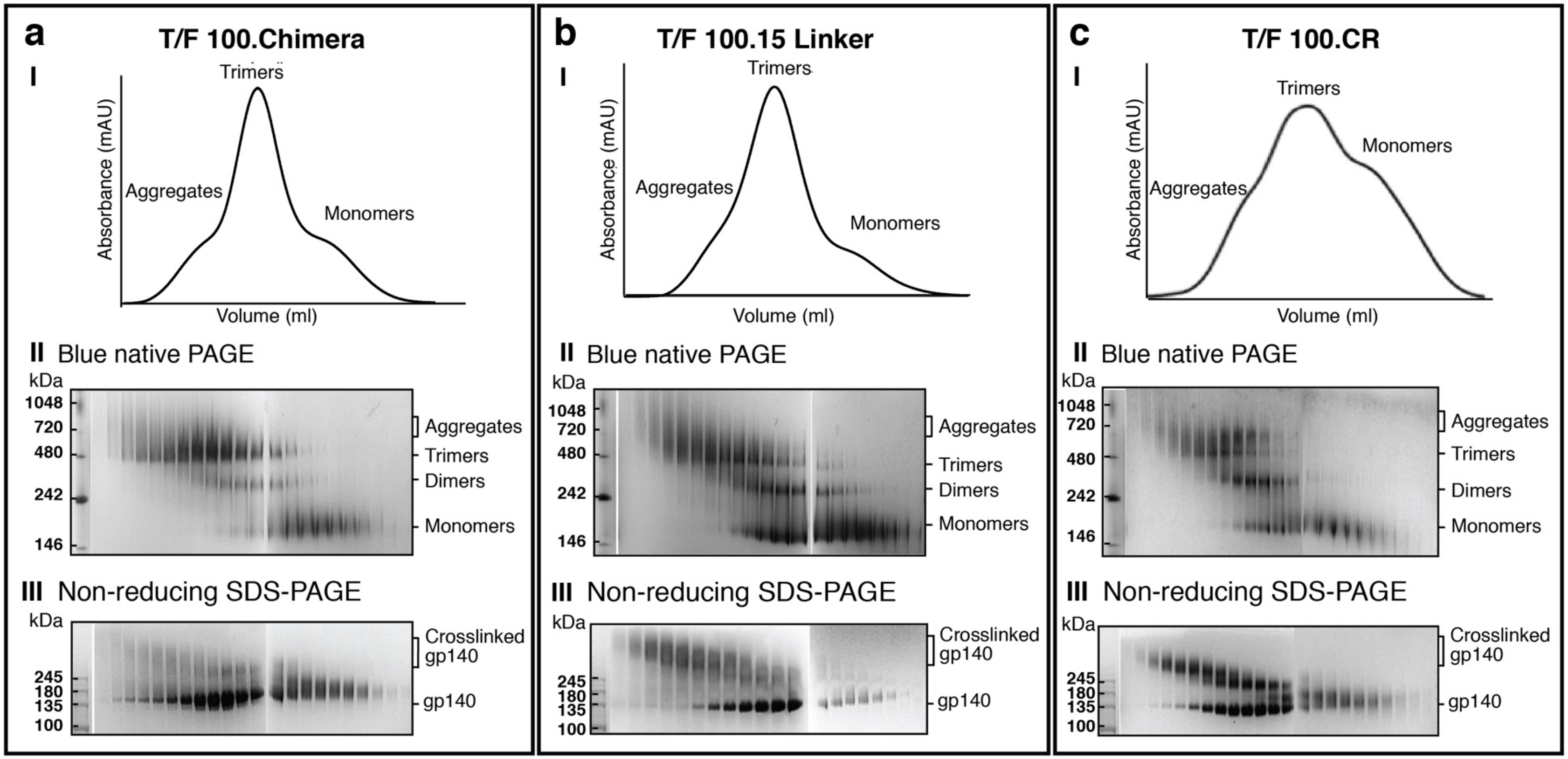

Supplementary Figure 2. Linker and Chimera trimers show significant amounts of aggregated and crosslinked gp140. **(I)** Elution profiles of: **a** T/F100.Chimera; **b** T/F100.15 Linker gp140; and **c** T/F100.CR gp140. CR represents cleavage resistant gp140 containing the furin cleavage-resistant sequence SEKS in place of the cleavage-sensitive sequence REKR[1]. The gp140s were purified by StrepTactin affinity chromatography and loaded on a Superdex 200 26/60 size exclusion column. **(II)** Blue native and **(III)** Non-

reducing PAGE of the eluted fractions. A slight gap was introduced between lanes when an image was sliced and pasted. The molecular masses in kDa are shown on the left. All the gels were stained with Coomassie Blue dye. See Methods for more details.

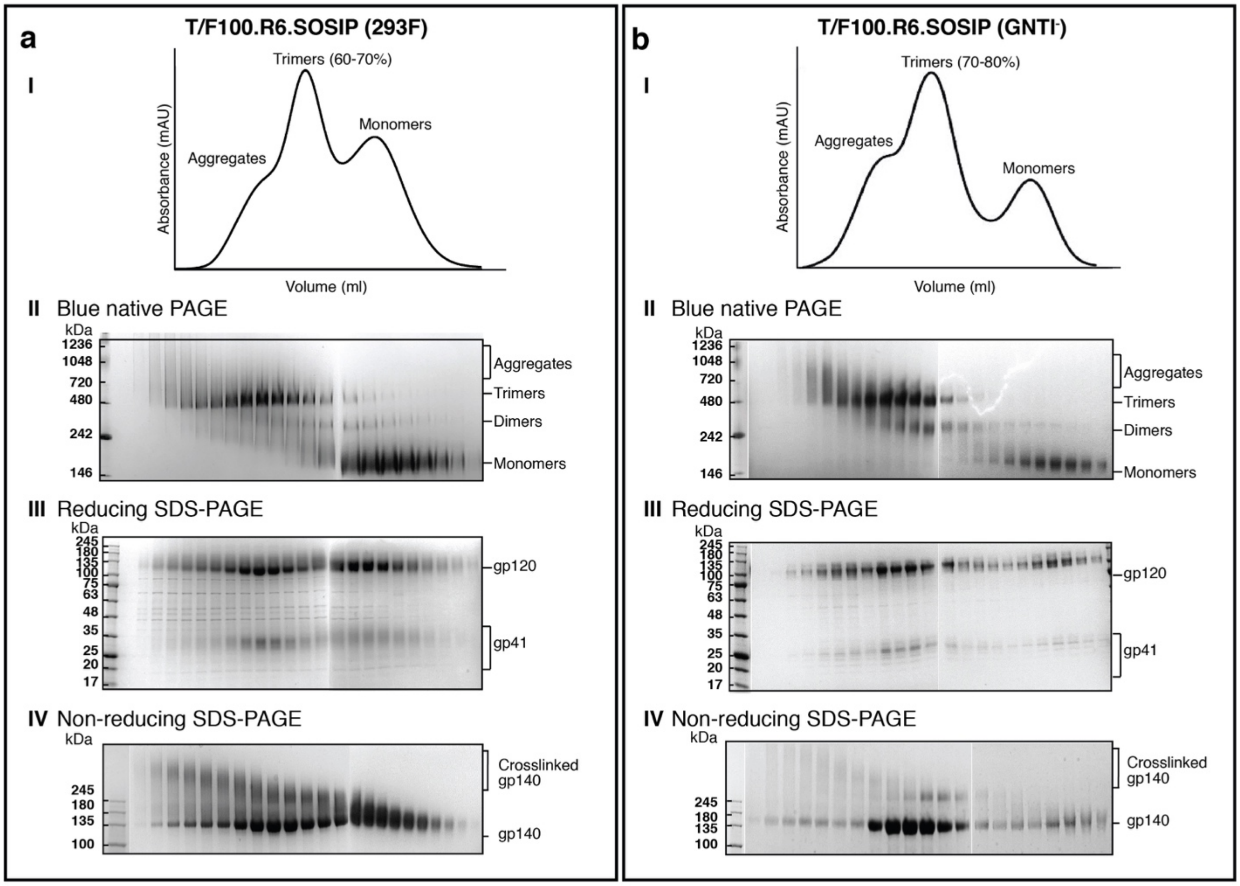

Supplementary Figure 3. Cleaved native-like T/F100 trimers produced in GNTI$^-$ cells were more homogeneous. **a** T/F100 gp140 produced in 293F cells. **b** T/F100 gp140 produced in GNTI$^-$ cells. **(I)** Elution profiles on a Superdex 200 26/60 size exclusion column. The gp140s were purified by StrepTactin affinity chromatography and loaded on the size exclusion column. **(II)** Blue native, **(III)** reducing, and **(III)** non-reducing PAGE of the eluted fractions. Note that the GNTI$^-$ produced trimers form relatively compact bands and contain less aggregates and/or crosslinked oligomers when compared to 293F produced trimers (compare panel IV of 293F fractions (**a**) to the same fractions of GNTI$^-$ (**b**)). A slight gap was introduced between lanes when an image was sliced and pasted. The molecular masses in kDa are shown on the left. All the gels were stained with Coomassie Blue dye. See Methods for more details.

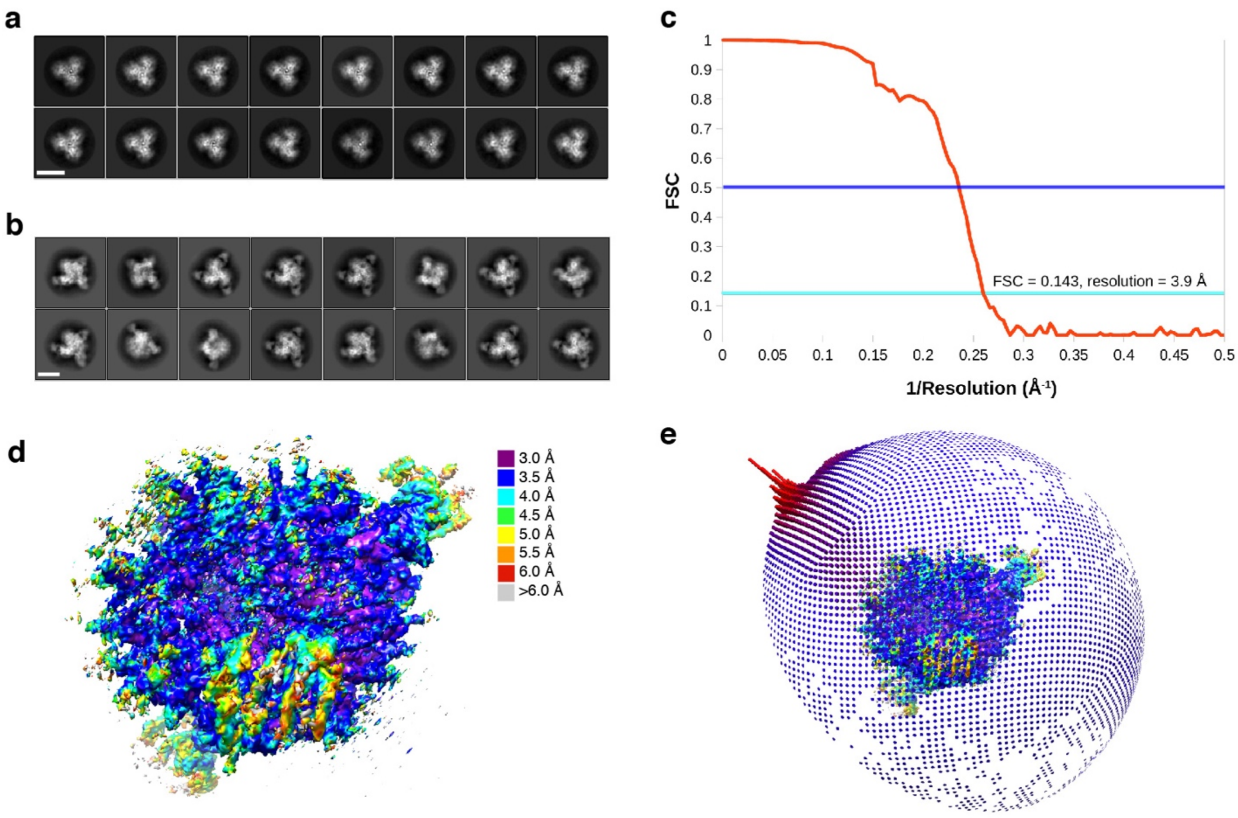

Supplementary Figure 4. Cryo-EM statistics. **a** Cryo-EM 2D class averages of the unliganded T/F100 trimers. **b** Cryo-EM 2D class averages of the T/F100 trimer in complex with the Fab fragment of the bNAb 8ANC195. **c** Gold-standard FSC curve, **d** local resolution estimate[2] and **e** angular distribution plot for the 3D cryo-EM reconstruction of the T/F100 trimer in complex with the Fab fragments of the bNAb 8ANC195. Scale bars, 100 Å.

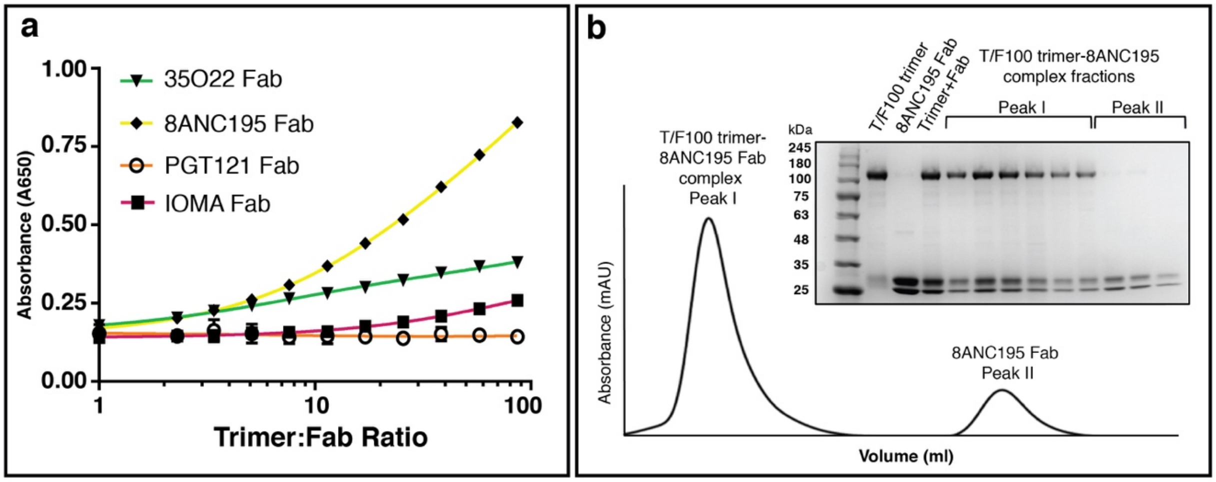

Supplementary Figure 5. The 8ANC195 broadly neutralizing antibody shows stronger binding to T/F100 trimers. **a** ELISA results comparing the binding of Fabs of various bNAbs to T/F100 trimers. The trimer:Fab ratio represent the molar ratio of trimers to Fab molecules. Results shown are representative of two independent experiments done in triplicates. Error bars denote standard deviation. **b** Size exclusion elution profile of the T/F100-8ANC195 Fab complex. Inset shows the SDS-PAGE of T/F100 trimer-8ANC195 Fab complex fractions (Peak 1). Molecular masses in kDa of the standard marker proteins are shown on the side of the gel.

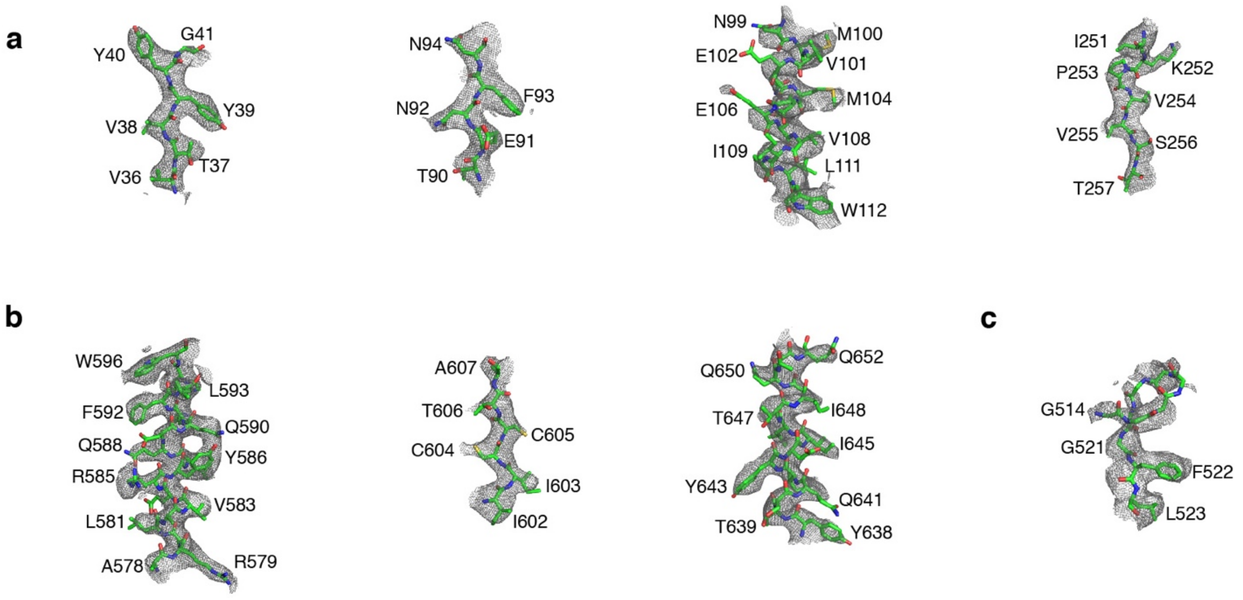

Supplementary Figure 6. Representative regions of the 3D cryo-EM reconstruction showing the T/F100 trimer in complex with the Fab fragments of the bNAb 8ANC195. **a** gp120. **b** gp41. **c** the fusion peptide region. Because residues 515-520 do not have reliable side-chain information, side chains of these residues were not modeled.

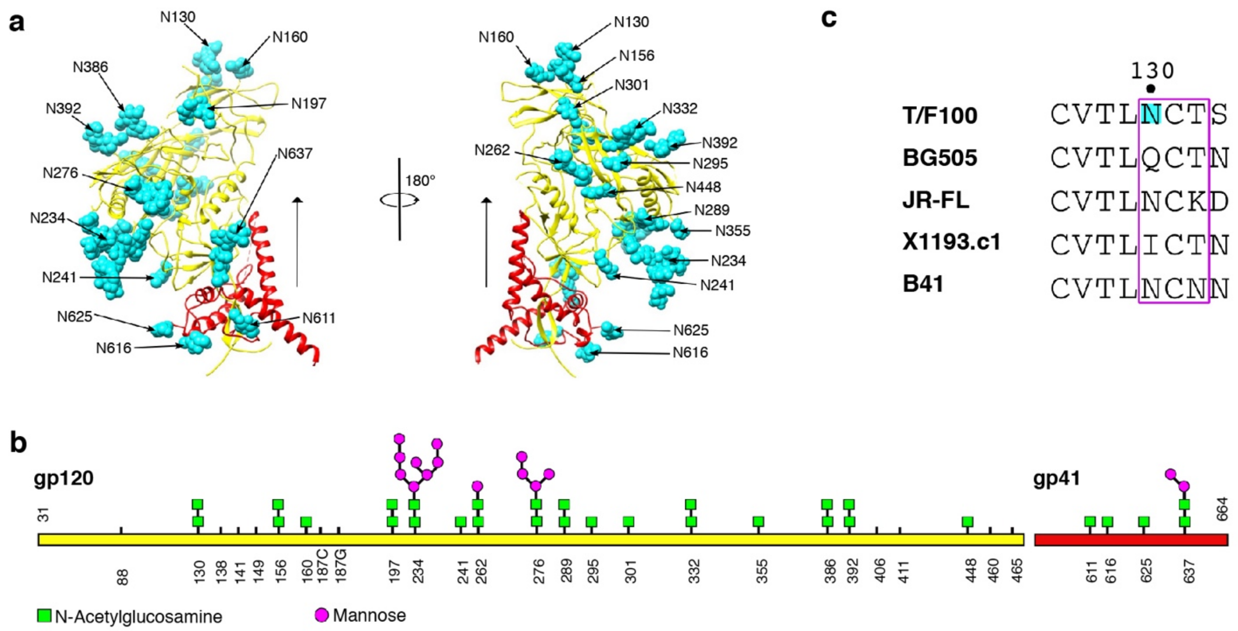

Supplementary Figure 7. N-linked glycosylation of the T/F100 trimer. **a** The visible N-linked glycans of one protomer in the structure of the T/F100 trimer. gp120 and gp41 are represented by ribbon diagrams, and colored yellow and red, respectively. N-linked glycans are represented by cyan spheres. **b** Schematic of N-linked glycans with all the potential N-linked glycosylation sites labeled and all the resolved sugar moieties shown. **c** The amino acid sequence around N130 of the T/F100 trimer is aligned to corresponding sequences of HIV-1 trimer structures from other strains. The potential N-linked glycosylation site of the T/F100 molecule is highlighted in cyan.

Figure 1c

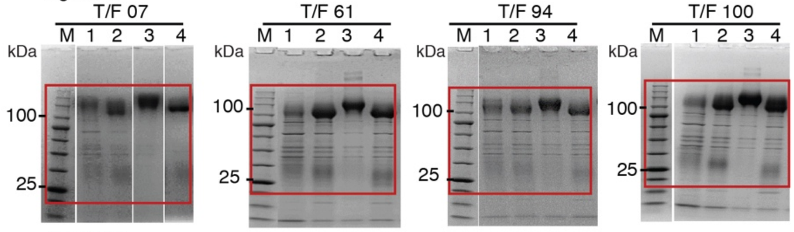

Figure 1d

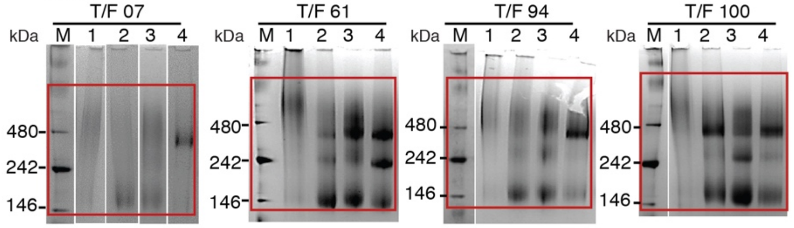

Figure 1e

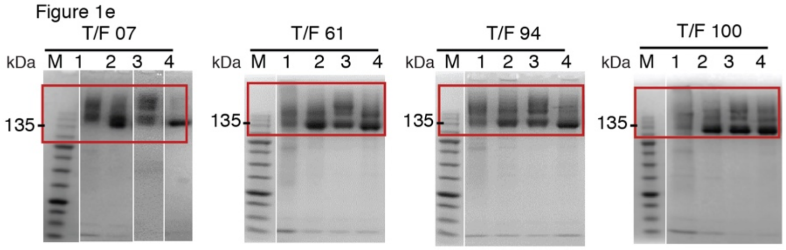

Supplementary Figure 2a

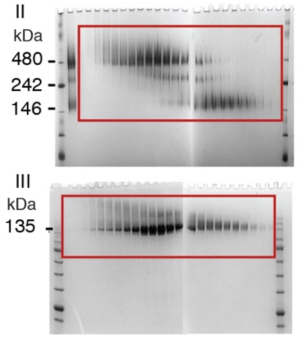

Supplementary Figure 2b

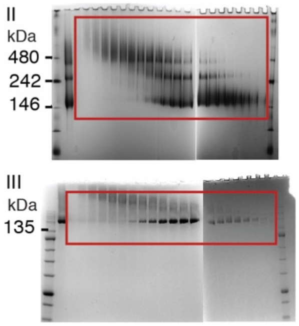

Supplementary Figure 2c

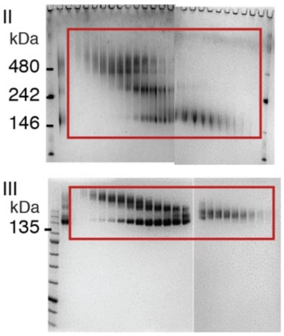

Supplementary Figure 3a

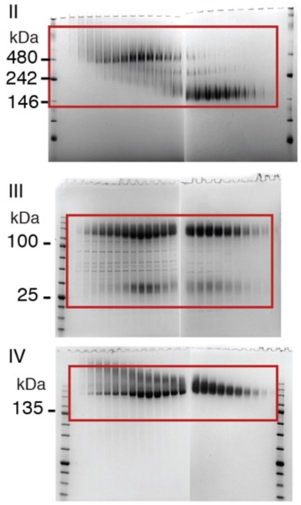

Supplementary Figure 3b

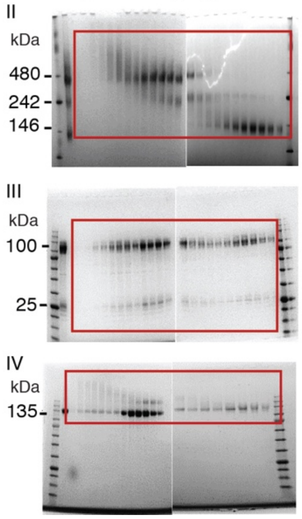

Supplementary Figure 5

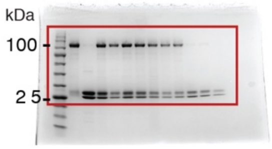

Supplementary Figure 8. Uncropped protein gels of various gp140 Env constructs. All the uncropped protein gels of the gp140 constructs shown in various figures in the main paper and supplementary information are provided in this figure. Red boxes in each panel correspond to the lanes shown in the respective figures.

Supplementary Table 1

Biochemical characterization of T/F CRF01_AE gp140 protomers

T/F gp140	Construct	Monomers	Dimers	Trimers	Aggregates	Non-Specific Crosslinking
**T/F07**	WT	–	–	–	++++	+++
	SOSIP	+++	–	+	++	++
	15Linker	+	+	++	+++	+++
	Chimera	–	–	+++	++	++
**T/F61**	WT	–	–	–	++++	+++
	SOSIP	+++	+	+	++	++
	15Linker	++	+	++	+++	+++
	Chimera	+	+++	+++	++	++
**T/F94**	WT	–	–	–	++++	++
	SOSIP	+++	+	+	++	++
	15Linker	++	++	++	+++	+++
	Chimera	+	–	+++	++	++
**T/F100**	WT	–	–	–	++++	+++
	SOSIP	++	+	++++	+	+
	15Linker	+++	++	+	+++	+++
	Chimera	+	+	+++	++	++

Supplementary Table 2

RMSD between equivalent Cα atoms of the T/F100 trimer and the other 15 HIV-1 Env trimer structures in the prefusion closed state (Comparisons were made with the T/F100 trimer superimposed onto each of the other trimers.)

PDB accession no.	Strain	RMSD (Å)	Number of equivalenced atoms
4TVP	BG505	1.5	1542
4ZMJ	BG505	1.5	1530
5ACO	BG505	1.8	1512
5CEZ	BG505	1.5	1533
5CJX	BG505	1.5	1458
5D9Q	BG505	1.5	1527
5FYJ	X1193.c1	1.6	1536
5FYK	JR-FL	1.6	1524
5FYL	BG505	1.5	1554
5I8H	BG505	1.5	1533
5T3X	BG505	1.7	1512
5T3Z	BG505	1.6	1524
5UM8	16055	1.5	1560
5V8L	BG505	1.7	1536
5V8M	BG505	1.7	1509

Supplementary Table 3

Codon-optimized gp140 Env DNA sequences

(T/F) CRF01_AE HIV-1 gp140 envelope	Codon Optimized gp140s
40007	CGCCAGCGACAACCTGTGGGTCACCGTGTACTACGGCGTGCCCGTGTGGCGGGATGCCGATACCACACTGTTC<br>TGTGCCAGCGACGCCAAGGCCCACGAAACCGAGGCCCATAATGTGTGGGCCACCCACGCCTGCGTGCCCACC<br>GATCCTAACCCCCAGGAAATCCACCTGGAAAACGTGACCGAGAACTTCAACATGTGGAAGAACAACATGGTG<br>GAACAGATGCAGGAAGATGTGATCAGCCTGTGGGACCAGAGCCTGAAGCCCTGCGTGAAGCTGACCCCTCTG<br>TGCGTGACCCTGAACTGCTCCAGCAACATCACCGTGCGGAACATCACAATCGCCAACGACACCTACGACATC<br>GGCAATATCACCGACGAGGCCAAGAATTGCAGCTTCAATATGACCACCGAGATCAAGGACAAGAAACAGCG<br>GGTGCACGCCCTGTTCTACAAGCTGGACATCGTGCCCATCAAGGATAACAACAACGACAGCGTGGAATACCG<br>GCTGATCAACTGCAACACCAGCGTGATCAAGCAGGCCTGTCCCAAGATCAGCTTCGACCCCATCCCCATCCAC<br>TACTGCACCCCTGCCGGCTACGCCATCCTGAAGTGCAACGAGAAGAATTTCAACGGCAAGGGCCCCTGCAAG<br>AACGTGTCCAGCGTGCAGTGTACCCACGGCATCAAGCCCGTGGTGTCCACCCAGCTGCTGCTGAATGGCAGC<br>CTGGCCGAGGAAGAGATCATCATCAGAAGCGAGAACCTGACCGACAACGCCAAGACCATCATCGTGCACCTG<br>AACGAGTCCGTGGAAATCAATTGCACCCGGCCCTCCAACAACACCCGGACCGGAATCCACATCGGCCCAGGC<br>CAGGTGTTCTACCGGACCGGCGACATCATCGGCAACATCCGGCAGGCCCACTGCAAAATCAACGGCACCAAG<br>TGGCGGAAGGTGCTGAAACAGGTCGTGGGCAAGCTGAGAGAGCACTTCGAGAACAAGAACATCACCTTCCA<br>GCCCCCCTCTGGCGGCGACCTGGAAATCACCATGCACCACTTCAACTGTCGGGGCGAGTTCTTCTACTGCAAT<br>ACCACCCGGCTGTTCAACACCACCAGACTGTTTAACAACACCTACAACAACCAGACCTGCAACGATACCATC<br>ATCCTGCCTTGCAAGATTAAGCAGATCATCCGCATGTGGCAGGAAGTGGGCCAGGCTATGTACGCCCCTCCTA<br>TCAGCGGCCCCATCGTGTGCGTGTCCAAGATCACCGGCATCCTGCTGACCAGAGATGGCGGCGTGAACAACA<br>CCGGCAACGAGACATTCAGACCCGGCGGAGGCAACATTAAGGACAACTGGCGGAGCGAGCTGTACAAGTAC<br>AAGGTGGTGCAGATCGAGCCCCTGGGAATCGCCCCTACCAAGTGCAAGAGAAGAGTGGTGCAGAGAAGGCG<br>GCGCAGACGGGCCGTGGGCATCGGAACAATGATCTTCGGCTTCCTGGGCGCTGCCGGCTCCACAATGGGAGC<br>CGCTTCTCTGACCCTGACCGTGCAGGCTAGACAGCTGCTGAGCGGCATCGTGCAGCAGCAGAGCAACCTGCT<br>GAGAGCCCCTGAGGCCCAGCAGCATCTGCTCCAGCTGACCGTGTGGGGCATCAAGCAGCTCCAGGCCAGAGT<br>GCTGGCCGTGGAAAGATACCTCCAGGACCAGCGGCTGCTGGGACTGTGGGGCTGTAGCGGCAAGATCATCTG<br>TTGCACCGCCGTGCCCTGGAACAGCACCTGGTCCAACAAGAGCTACAACGAGATTTGGAGCAACATGACCTG<br>GACCCAGTGGGAGAAAGAGATCAGCAACTACACCGCCCAAATCTATGACATCCTGACCGAGAGCCAGAGCC<br>AGCAGGATAAGAACGAGAAGGACCTGCTGGAACTGGAC
40061	CGCCAGCGACAATCTGTGGGTCACCGTGTACTACGGCGTGCCCGTGTGGAAGGACGCCGACACCACACTGTT<br>CTGTGCCAGCGACGCCAAGGCCCACTCTACCGAGGCCCATAATGTGTGGGCCACCCACGCCTGCGTGCCCAC<br>CGATCCTAACCCCCAGGAAATCCACCTGGAAAACGTGACCGAGAACTTCAATATGTGGAAGAACAACATGGT<br>GGAACAGATGCAGGAAGATATTATCAGCCTGTGGGACCAGAGCCTCCAGCCCTGCGTGAAGCTGACCCCTCT<br>GTGCGTGACCCTGAACTGCACCAACGCCAACGCCACCAACGTGAACATCCCCGAGAACCTGAACCTGAAGAA<br>CATCGGCAACATCACCGACGACCTGCGGAACTGCTCCTTCAACATGACCACCGTGCTGCGGGACAAGAAAAA<br>ACAGGTGCACGCCCTGTTCTACCGGCTGGACATCGTGGAAATGGAAATCAACAACAGCACCAAGTACCGGCT<br>GATCAACTGCAACACCAGCGTGATCAAGCAGGCCTGTCCCAAGATCAGCTTCGACCCCATCCCCATCCACTAC<br>TGCACCCCTGCCGGCTACGCCATCCTGAAGTGCAACGACAAGAATTTCAACGGCACCGGCCCCTGCAAGAAT<br>GTGTCCAGCGTGCAGTGCACCCACGGCATCAAGCCTGTGGTGTCCACCCAGCTGCTGCTGAATGGCAGCCTGG<br>CCGAGGAAGAGATCATCATCAGAAGCGAGAATCTGACCGACAACGCCAAGACCATCATTGTGCACCTGAACA<br>AGAGCGTGGAAATCAATTGCACCCGGCCCAGCAACAACACCAGAACCAGCATGACCATCGGCCCAGGCCAG<br>GTGTTCTACAGAACCGGCGAGATCATCGGCAATATCCGGCAGGCCTACTGCGAGATCAACGAGACAAAGTGG<br>AACGAGACACTGAAACAGGTGGCCGCCAAGCTGAAAGAGCACTTCAACCGGACCATCCGGTTCCAGCCTCCC<br>TCTGGCGGCGACCTGGAAATCACCATGCACCACTTCAACTGTCGGGGCGAGTTCTTCTACTGCAATACCACCA<br>AGCTGTTCAACCACACATGCGAGGGCAACGAGGGCTGCAATGGCACCAGCAGCAACGGCACAATCATCCTGC<br>CTTGCAGAATCAAGCAGATCATCAATATGTGGCAGGGCGCTGGCCAGGCTATGTACGCCCCTCCTATCCGGG<br>GCACCATCAACTGCGTGTCCAATATCACCGGCCTGCTGCTGACCAGGGACGGCGGCAAGGATAATAGCACCA<br>GCGGCAATGAGACATTCAGACCCGGCGGAGGCAATATCAAGGACAACTGGCGGAGCGAGCTGTACAAGTAC<br>AAGGTGGTGCAGATCGAGCCCCTGGGAATCGCCCCTACCAGAGCCAAGAGAAGAGTGGTGCAGCGCAGGCG<br>ACGGCGCAGAGCCGTGGGCATCGGCGCTATGCTGTTCGGCTTTCTGGGAGCCGCCGGAAGCACAATGGGCGC<br>TGCCAGCATCACCCTGACCGTGCAGGCTAGACAGCTGCTGAGCGGCATCGTGCAGCAGCAGAGCAACCTGCT<br>GAGAGCCATTGAGGCCCAGCAGCATCTGCTCCAGCTGACCGTGTGGGGCATTAAGCAGCTCCAGGCCAGAGT<br>GCTGGCCGTGGAAAGATACCTGAAGGACCAGAAATTCCTGGGACTGTGGGGCTGCTCTGGCAAGATCATCTG<br>CACAACCGCCGTGCCTTGGAACAGCACCTGGTCCAACAAGAGCTACAACGAGATTTGGGACAATATGACCTG<br>GATCGAGTGGGAGAGAGAGATCAGCAATTACACCAACCAAATCTACGAGATCCTGACCGAGAGCCAGAATC<br>AGCAGGACCGGAACGAGAAGGATCTGCTGGAACTGGAC
40094	CGCCAGCAACAATCTGTGGGTCACCGTGTACTACGGCGTGCCCGTGTGGCGGGATGCCGATACCACACTGTTC<br>TGTGCCAGCGACGCCAAGGCCCACGAGACAGAGATCCACAATGTGTGGGCCACCCACGCCTGCGTGCCCACC<br>GATCCTAACCCCCAGGAAATCCCCCTGAAGAACGTGACCGAGAACTTCAATATGTGGAAGAACAACATGGTG<br>GAACAGATGCAGGAAGATGTGATCAGCCTGTGGGACCAGAGCCTGAAGCCCTGCGTGAAGCTGACCCCTCTG

	TGCGTGACCCTGCACTGCACCAAAGTGACCCTGAACGCCAACGGCACCAACAACGCCACCACCAACAATACC<br>ATCACCAGAGCCAACGTGACCACCGGCATCAATGTGTCCAACATCACCGAGGAAATCCGGAACTGCTCCTTC<br>AATATCACCACCGAAGTGCGGGACAAGCAGAAACAGGTGCACGCCCTGTTCTACCGGCTGGACATCGTGCCC<br>ATCGACAGCAACAACAACTCCAACAAGAACAGCAACCGCGAGTACCGGCTGATCAACTGCAACACCAGCGT<br>GATCAAGCAGGCCTGTCCCAAGGTGTCCTTCGACCCCATCCCCATCCACTACTGTACCCCTGCCGGCTACGTG<br>ATCCTGCGGTGCAACGAGAAGAATTTCAACGGCGCTGGCCCTTGCAAGAATGTGTCCTCCGTGCAGTGTACCC<br>ACGGCATCAAGCCCGTGGTGTCCACCCAGCTGCTGCTGAATGGCAGCCTGGCCGAAGAGGAAATCGTGATCA<br>GAAGCGAGAACCTGACCAACAATGCCAAGACCATCATTGTGCACCTGAACGAGAGCGTGACCATCAATTGCA<br>CCCGGCCCTCCAACAACACCCGGACCAGCATCAGAATCGGCCCAGGCCAGGTGTTCTACCAGACCGGCCAGA<br>TCACCGGCGACATCCGGAAGGCCTATTGCGAGGTGGACGAGGCCAAGTGGAACAAGACCCTGAAACAGGTG<br>GCCGAGAAGCTGAAGAAGCACTTCAACAGCACCACCATCATCTTCCAGCCCCCCTCTGGCGGCGACCTGGAA<br>ATCACCACCCTGCATTTCAACTGTCGGGGCGAGTTCTTCTACTGCAATACCACCAGCCTGTTCAACAAGACCT<br>ACATCGGCAACGAGACACGGAACGAAACCCACGACGGCGGCAGCAACAAGACAATCACCCTGCCCTGCAAG<br>ATTAAGCAGATCGTGCGGATGTGGCAGGGCGTGGGCCAGGCTATGTATGCCCCTCCTATCAGCGGAGTGATC<br>AATTGCGTGTCCAATATTACCGGCATCCTGCTGACCAGGGACGGCGGACAGGGCGGCCACAACAACACAACC<br>GAGACAGAAACCTTCAGACCCGGCGGAGGCAACATCAAGGACAACTGGCGGAGCGAGCTGTACAAGTACAA<br>GGTGGTGCAGATCGAGCCCCTGGGAATCGCCCCCACAAGAGCCAAGCGGCGGGTGGTGGAACGCAGGCGAC<br>GGCGCAGAGCCGTGGGCATCGGCGCTATGATCTTCGGCTTTCTGGGAGCCGCCGGAAGCACAATGGGCGCTG<br>CCAGCATCACCCTGACCGTGCAGGCTAGACAGCTGCTGAGCGGCATCGTGCAGCAGCAGAGCAACCTGCTGA<br>GAGCCATCGAGGCCCAGCAGCATCTGCTCCAGCTGACCGTGTGGGGAATCAAGCAGCTCCAGGCCAGAGTGC<br>TGGCCGTGGAAAGATACCTGAAGGACCAGAAATTCCTGGGACTGTGGGGCTGCTCCGGCAAGATCATCTGCA<br>CAACCGCCGTGCCTTGGAACAGCACCTGGTCCAACAGAAGCTATGAGGAAATTTGGAACAACCTGACCTGGG<br>TGGAATGGGAGAGAGAGATCAGCAACTACACCAACCAAATCTACGCCATCCTGACCGAGAGCCAGAACCAG<br>CAGGACCGGAACGAGAAGGACCTGCTGGAACTGGAC
40100	CGCCACCAACAATCTGTGGGTCACCGTGTACTACGGCGTGCCCGTGTGGCGGGATGCCGATACCACACTGTTC<br>TGTGCCAGCGACGCCAAGGCCCACGAGACAGAGGTGCACAATGTGTGGGCCACCCACGCCTGCGTGCCCACC<br>GATCCTAACCCCCAGGAAATGCACCTGAAGAACGTGACCGAGAACTTCAATATGTGGAAGAACAACATGGTG<br>GAACAGATGCAGGAAGATGTGATCAGCCTGTGGGACCAGAGCCTGAAGCCCTGCGTGAAGCTGACCCCTCTG<br>TGCGTGACCCTGAACTGCACCAGCGCCACCGTGACCAACTACACCAAAGTGAACGACACCAGCGACATCATC<br>GGCAACATCACCGACGACGTGCGGAACTGCTCCTTCAACATGACCACCGAGCTGCGGGACAAGCAGCAGAAG<br>GTGTACGCCCTGTTCTACAAGCTGGACATCGTGCCCATCGACGACAGCAGCAACAACGGCAGCTCCAACTTC<br>AGCGAGTACCGGCTGATCAACTGCAACACCAGCGTGATCAAGCAGGCCTGTCCCAAGGTGTCCTTCGACCCC<br>ATCCCCATCCACTACTGTACCCCTGCCGGCTACGCCATCCTGCGGTGCAACGACAAGAAGTTCAACGGCACCG<br>GCCCTTGCAAGAATGTGTCCAGCGTGCAGTGCACCCACGGCATCAAGCCTGTGGTGTCCACCCAGCTGCTGCT<br>GAATGGCAGCCTGGCCGAGGAAGGCATCATCATCAGAAGCGAGAACCTGACCAACAACGCCAAGACCATCA<br>TCGTGCACTTCAACGAGAGCGTGAAGATCAATTGCACCCGGCCCTCCAACAACACCCGGACCGGAATCCACA<br>TCGGCCCAGGCCAGGTGTTCTATAAGACCGGCGATATTATCGGCGACATCCGGAAGGCCTACTGCAACATCTC<br>TGGCGCCCAGTGGCACAAGGTGCTGGGCAGAGTGGCCAACAAGCTGAAAGAGCACTTTAACAACAAGACAA<br>TCGTGTTCAAGCCCAGCTCTGGCGGCGACCCCGAGATCACCATGCACCACTTCAACTGTCGGGGCGAGTTCTT<br>CTACTGCAATACCACCAAGCTGTTCAACAGCACCTGGGGCGGCAACAAGAACGAGACACGGGACAACGGAA<br>CCATCACCATCCCTTGCAGAATCAAGCAGATCATCAATATGTGGCAGGGCGTGGGCCAGGCTATGTACGCCC<br>CTCCTATCAAGGGCGTGATCAAATGCCTGAGCAATATCACCGGCATCCTGCTGACCAGGGACGGCGGCAATG<br>ACAGCACCGAGAACAACGAGACATTCAGACCCGGCGGAGGCAATATCAAGGACAACTGGCGGAACGAGCTG<br>TACAAGTACAAGGTGGTGCAGATCGAGCCCCTGGGAATCGCCCCTACCAAGTGCAAGCGGCGGGTGGTGGAA<br>CGCAGGCGACGGCGCAGAGCCGTGGGCCTGGGAGCTATGATCTTCGGCTTTCTGGGAGCCGCCGGAAGCACA<br>ATGGGCGCTGCCAGCATCACCCTGACCGTGCAGGCTAGACAGCTGCTGAGCGGCATCGTGCAGCAGCAGAGC<br>AACCTGCTGAGAGCCCCGGAGGCCCAGCAGCATCTGCTCCAGCTGACCGTGTGGGGCATCAAACAGCTCCAG<br>GCCAGAGTGCTGGCCGTGGAAAGATACCTCCAGGACCAGAAATTCCTGGGACTGTGGGGCTGCTCCGGCAAG<br>ATCATCTGTTGCACCGCCGTGCCTTGGAACAGCTCCTGGTCCAACAAGACCTTCGAAGAGATTTGGAACAATA<br>TGACCTGGATCGAGTGGGAGCGCGAGATCAGCAATTACACCAGCCAAATCTACGACATCCTGACCATCAGCC<br>AGACCCAGCAGGAAAAGAACGAAAAGGACCTGCTGGAACTGGAC

Supplementary Table 4

Primers used for construction of the gp140 Envs

gp140 Primers	Forward(F) /Reverse (R)	Nucleotide sequences 5'- to -3'
Terminal T/F 07.R6	F	CTAGCTAGCTGCCAGCGACAACCTGTGGGTCACCGT
Terminal T/F 61.R6	F	CTAGCTAGCCGCCAGCGACAATCTGTGGGTCACCG
Terminal T/F 94.R6	F	CTAGCTAGCCGCCAGCAACAATCTGTGGGTCACCG
Terminal T/F100.R6	F	CTAGCTAGCCGCCACCAACAATCTGTGGGTCACCG
Terminal T/F (07, 61, 94, 100).R6	R	TTTTCCTTTTGCGGCCGCGTCCAGTTCCAGCAG
BG505	F	CTAGCTAGCTGCCGAGAACCTGTGGGTCACCGTGTA
	R	TTTTCCTTTTGCGGCCGCGTCCAGGGCCAGCAGGTC
T/F 07. 15Linker	F	CCAAGAGAAGAGTGGTGCAGGGCGGAAGTGGAGGCGGCGGCAGCGGGGGAGG TGGCTCCGGAGGAGCCGTGGGCATCGGAACAAT
	R	ATTGTTCCGATGCCCACGGCTCCTCCGGAGCCACCTCCCCCGCTGCCGCCGCCTCC ACTTCCGCCCTGCACCACTCTTCTCTTGG
T/F 07. Chimera	F	CAAGAGAAGAGTGGTGCAGCGCAGGCGACGGCGCAGAGCCGTGGGAATTGGAG CTGT
	R	ACAGCTCCAATTCCCACGGCTCTGCGCCGTCGCCTGCGCTGCACCACTCTTCTCTTG
T/F 61. 15Linker	F	CAAGAGAAGAGTGGTGCAGGGCGGAAGTGGAGGCGGCGGCAGCGGGGGAGGTG GCT CCGGAGGAGCCGTGGGCATCGGCGCTAT
	R	ATAGCGCCGATGCCCACGGCTCCTCCGGAGCCACCTCCCCCGCTGCCGCCGCCTCC ACTTCCGCCCTGCACCACTCTTCTCTTG
T/F 61. Chimera	F	CAAGAGAAGAGTGGTGCAGCGCAGGCGACGGCGCAGAGCCGTGGGAATTGGAG CTGT
	R	ACAGCTCCAATTCCCACGGCTCTGCGCCGTCGCCTGCGCTGCACCACTCTTCTCTTG
T/F 94. 15Linker	F	GCAAGCGGCGGGTGGTGGAAGGCGGAAGTGGAGGCGGCGGCAGCGGGGGAGGT GGCTCCGGAGGAGCCGTGGGCATCGGCGCTAT
	R	ATAGCGCCGATGCCCACGGCTCCTCCGGAGCCACCTCCCCCGCTGCCGCCGCCTCCA CTTCCGCCTTCCACCCGCCGCTTG
T/F 94. Chimera	F	GCAAGCGGCGGGTGGTGGAACGCAGGCGACGGCGCAGAGCCGTGGGAATTGGAG CTGT
	R	ACAGCTCCAATTCCCACGGCTCTGCGCCGTCGCCGTCGTTCCACCACCCGCCGCTTGC
T/F 100. 15Linker	F	GCAAGCGGCGGGTGGTGGAAGGCGGAAGTGGAGGCGGCGGCAGCGGGGGAGGT GGCTCCGGAGGAGCCGTGGGCCTGGGAGCTATGATCT
	R	AGATCATAGCTCCCAGGCCCACGGCTCCTCCGGAGCCACCTCCCCCGCTGCCGCCGC CTCCACTTCCGCCTTCCACCACCCGCCGCTTGC
T/F 100. 20Linker	F	GGGGAGGTGGCTCCGGAGGAGGCGGTAGCGGAGGGGCCGTGGGCCTGGGAGC TATG
	R	CATAGCTCCCAGGCCCACGGCCCCTCCGCTACCGCCTCCTCCGGAGCCACCTCCCC
T/F 100. CR	F	CAAGCGGCGGGTGGTGGAAAGCGAGAAGAGCGCCGTGGGCCTGGGAGCTATG
	R	CATAGCTCCCAGGCCCACGGCGCTCTTCTCGCTTTCCACCACCCGCCGCTTG
T/F 100. Chimera	F	TGCAAGCGGCGGGTGGTGGAACGCAGGCGACGGCGCAGAGCCGTGGGA
	R	ACAGCTCCAATTCCCACGGCTCTGCGCCGTCGCCTGCGTTCCACCACCCGCCGCTT GCA
BG505-T/F100FP	F	GTGGGAATTGGAGCTATGATCTTCGGCTTTCTGGGCGCTG
	R	CAGCGCCCAGAAAGCCGAAGATCATAGCTCCAATTCCCAC

Supplementary Table 5

Cryo-EM data collection, refinement and validation statistics

	T/F100 trimer-8ANC195 Fab complex (EMDB-0485) (PDB 6NQD)
**Data collection and processing**	
Magnification	29,000
Voltage (kV)	300
Electron exposure (e^{-}/Å^2)	64
Defocus range (μm)	1.5–3.5
Physical pixel size (Å)	1.0
Symmetry imposed	C3
Initial particle images (no.)	353,000
Final particle images (no.)	170,716
Map resolution (Å)	3.9
FSC threshold	0.143
**Refinement**	
Map sharpening B factor (Å^2)	-162
R.m.s. deviations	
Bond lengths (Å)	0.01
Bond angles (°)	1.44
Validation	
MolProbity score	2.96
Clashscore	14.7
Poor rotamers (%)	8.15
Ramachandran plot	
Favored (%)	88.35
Allowed (%)	10.88
Disallowed (%)	0.77

Supplementary References

1 AlSalmi, W. *et al.* A new approach to produce HIV-1 envelope trimers: both cleavage and proper glycosylation are essential to generate authentic trimers. *J Biol Chem* **290**, 19780-19795 (2015).

2 Kucukelbir, A., Sigworth, F. J. & Tagare, H. D. Quantifying the local resolution of cryo-EM density maps. *Nat Methods* **11**, 63-65 (2014).
